# Supplementary figures and images for: Frequency-dependent selection can forecast evolution in Streptococcus pneumoniae
Source: PLoS Biol. 2020 Oct 22;18(10):e3000878. doi: 10.1371/journal.pbio.3000878 (PMC7580979; doi:10.1371/journal.pbio.3000878)

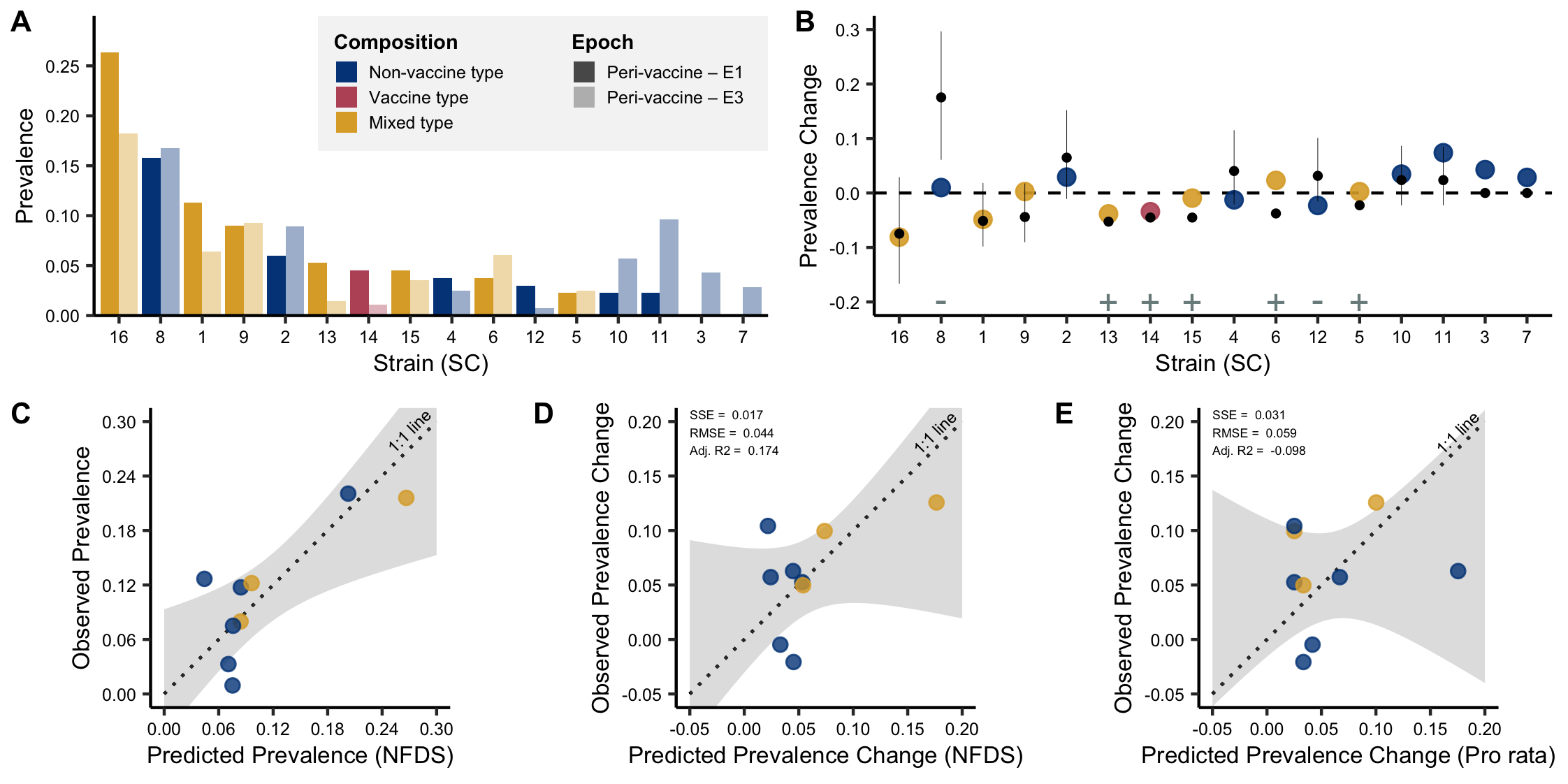

Supplement: S2 Fig — A, Peri-vaccine–E1 (2001) to Peri-vaccine–E3 (2007) change in prevalence of 16 strains (sequence clusters, SCs). Strains are ordered from highest to lowest pre-vaccine prevalence. B, Change in prevalence from Peri-vaccine–E1 to Peri-vaccine–E3 ordered by strain as in (A). Observed changes in prevalence are represented by points colored by serotype composition of the strain: nonvaccine type, PCV7 vaccine type, and mixed (vaccine and nonvaccine types). The point and whiskers show the prevalence change expected if all VT strains were removed and NVT increased pro rata to their pre-vaccine prevalence. The dot is the median, and the whiskers give the 2.5% and 97.5% quantiles of predicted changes under the null model using 10,000 bootstraps from pre-vaccine and post-vaccine samples. Significant differences are denoted with plus and minus signs specifying strains that were significantly more (n = 5) or less (n = 2) common, respectively, than expected under the null model. C, Scatterplot of observed versus predicted prevalence of 9 strains at post-vaccine equilibrium based on quadratic programming. These 9 strains contained at least one NVT strain pre-vaccine. Points are colored based on serotype composition as described in panel A. Perfect predictions would lie on the dotted line of equality (1:1 line). The shaded grey region shows the confidence interval from the linear regression model used to test for deviation of the observed versus predicted values compared to the 1:1 line. D–E, Comparison of the predicted prevalence change from quadratic programming analysis using accessory genes (D, p = 0.38; intercept 95% CI −0.08 to 0.03; slope 95% CI −0.24 to 1.32) and naive pro rata model (E, p = 0.07; intercept 95% CI −0.11 to 0.02; slope 95% CI −0.65 to 1.03) as shown in panel A. Goodness-of-fit statistics including SSE, RMSE, and degrees of freedom Adj. R2 are given for each model. The lower SSE indicates a better model fit. Panel A presents the frequencies of 16 strains base [file pbio.3000878.s002.tiff]

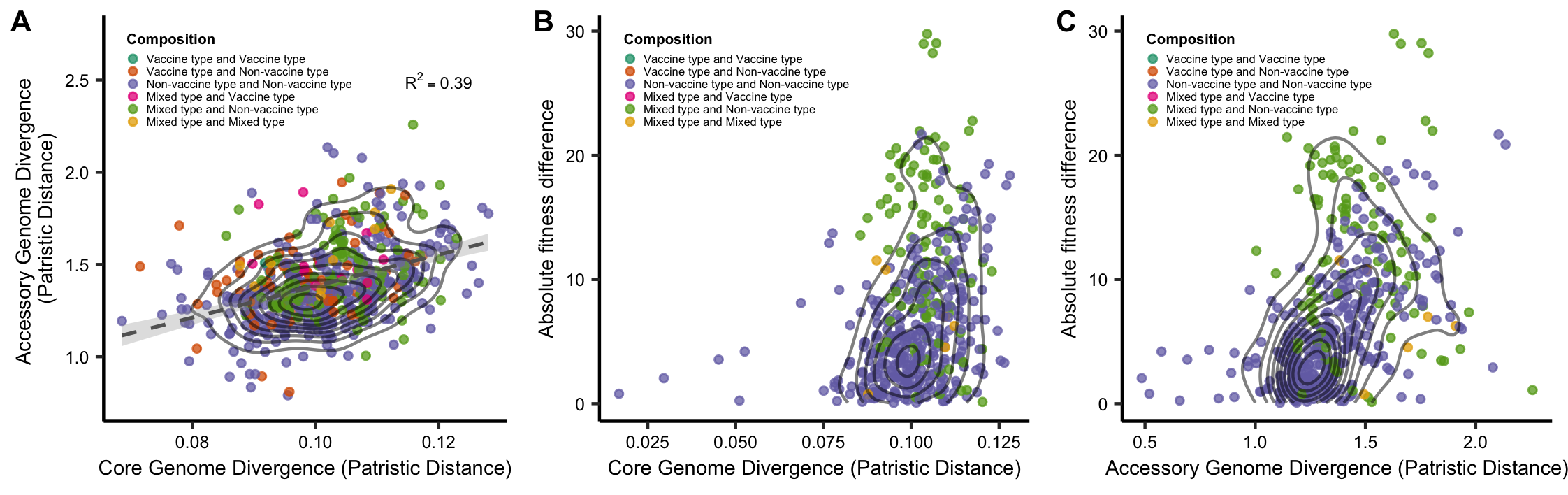

Supplement: S3 Fig — A, Comparison of core and accessory genome divergence, quantified as the mean patristic distance between strains (SCs) on respective core and accessory genome maximum likelihood phylogenies. Each point represents a pairwise comparison of the mean between-strain patristic distances. Points are colored by the vaccine type (VT, NVT, and mixed) of strains in the pairwise comparison. Pairwise comparisons between strain sub-clusters (e.g., SC-25A and SC-25B, see S1 Fig phylogeny) and between the unencapsulated nontypeable SC-01 and other strains have been excluded. This removes the most and least divergent pairwise comparisons, which otherwise artificially inflated the linear relationship between core and accessory genome distances. The dotted line represents the fit of a linear regression model to the data with 95% CI shaded in gray. Contour lines show 2-dimensional kernel density estimation based on the distribution of points. B, Core genome divergence (as defined here based on the core genomic phylogeny patristic distances) and the absolute fitness difference among 31 strains presented in Fig 3A. C, Accessory of core genome divergence (as defined here based on the core genomic phylogeny patristic distances) and the value of the fitness difference among 31 strains presented in Fig 3A. Of note, there is a considerable range in predicted fitness difference among strains that have similar accessory and core genome divergence (e.g., core genome divergence of 0.1 or accessory genome divergence of 1.0–1.5). See S1 Data and S1 Code for details. NVT, nonvaccine serotype; SC, sequence cluster; VT, vaccine serotype. (TIFF) [file pbio.3000878.s003.tiff]

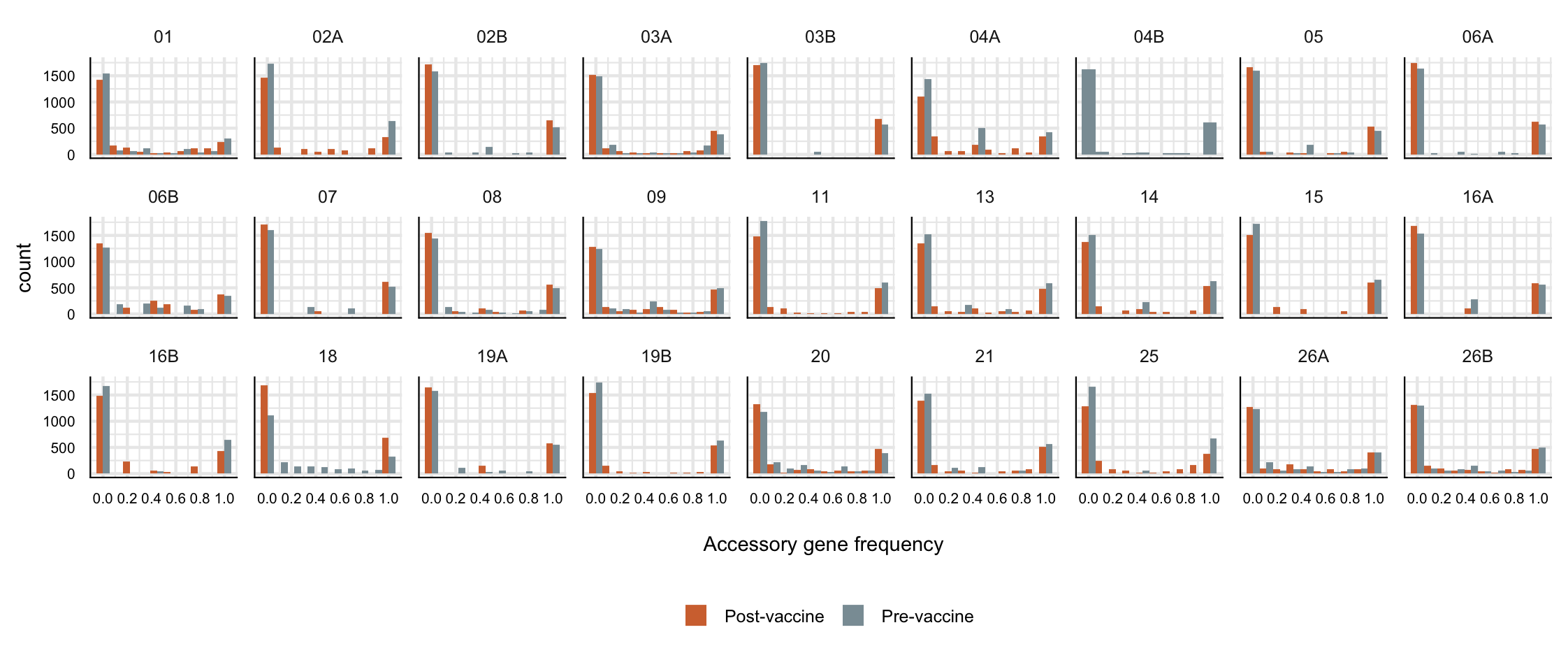

Supplement: S5 Fig — Histograms represent the presence and absence of accessory genes that were present in between 5% and 95% of the entire sample. Note, each strain will have a variable number of accessory genes depending on their accessory genome diversity. Within each strain, the distribution of accessory genes remained relatively stable from pre- to post-vaccine. See S1 Data and S1 Code for details. NVT, nonvaccine serotype. (TIFF) [file pbio.3000878.s005.tiff]
