# Supplementary material for: Frequency-dependent selection can forecast evolution in Streptococcus pneumoniae
Source: PLoS Biol. 2020 Oct 22;18(10):e3000878. doi: 10.1371/journal.pbio.3000878 (PMC7580979; doi:10.1371/journal.pbio.3000878)
Supplement: S1 Table — NVT, nonvaccine serotype; VT, vaccine serotype. (DOCX) [file pbio.3000878.s006.docx]

| **STRAIN** | **Pre-vaccine** | | | | **Perturbation (“Peri-vaccine”)** | | | | **Post-vaccine equilibrium** | | | |  | | |
| --- | --- | --- | --- | --- | --- | --- | --- | --- | --- | --- | --- | --- | --- | --- | --- |
|  | **NVT** | **VT** | **Total** | **Prevalence** | **NVT** | **VT** | **Total** | **Prevalence** | **NVT** | **VT** | **Total** | **Prevalence** | **Total** | **Prevalence** |  |
| 1 | 9 |  | 9 | 3.3% | 10 |  | 10 | 2.5% | 17 |  | 17 | 6.4% | 36 | 3.8% |  |
| 02A | 1 |  | 1 | 0.4% | 11 |  | 11 | 2.8% | 7 |  | 7 | 2.6% | 19 | 2.0% |  |
| 02B | 6 |  | 6 | 2.2% | 3 |  | 3 | 0.8% | 1 |  | 1 | 0.4% | 10 | 1.1% |  |
| 03A | 10 |  | 10 | 3.6% | 18 |  | 18 | 4.5% | 9 |  | 9 | 3.4% | 37 | 3.9% |  |
| 03B | 2 |  | 2 | 0.7% | 5 |  | 5 | 1.3% | 1 |  | 1 | 0.4% | 8 | 0.9% |  |
| 04A | 2 |  | 2 | 0.7% | 4 |  | 4 | 1.0% | 12 |  | 12 | 4.5% | 18 | 1.9% |  |
| 04B | 6 |  | 6 | 2.2% | 3 |  | 3 | 0.8% |  |  |  | 0.0% | 9 | 1.0% |  |
| 04C |  | 9 | 9 | 3.3% |  | 4 | 4 | 1.0% |  | 3 | 3 | 1.1% | 16 | 1.7% |  |
| 5 | 6 |  | 6 | 2.2% | 9 |  | 9 | 2.3% | 6 |  | 6 | 2.3% | 21 | 2.2% |  |
| 06A | 6 |  | 6 | 2.2% | 3 |  | 3 | 0.8% | 1 |  | 1 | 0.4% | 10 | 1.1% |  |
| 06B | 6 |  | 6 | 2.2% | 4 |  | 4 | 1.0% | 5 |  | 5 | 1.9% | 15 | 1.6% |  |
| 7 | 3 |  | 3 | 1.1% | 10 |  | 10 | 2.5% | 2 |  | 2 | 0.8% | 15 | 1.6% |  |
| 8 | 10 |  | 10 | 3.6% | 13 |  | 13 | 3.3% | 5 |  | 5 | 1.9% | 28 | 3.0% |  |
| 9 | 10 | 19 | 29 | 10.6% | 27 |  | 27 | 6.8% | 19 |  | 19 | 7.2% | 75 | 8.0% |  |
| 10 |  |  |  | 0.0% | 5 |  | 5 | 1.3% | 7 |  | 7 | 2.6% | 12 | 1.3% |  |
| 11 | 1 |  | 1 | 0.4% | 8 |  | 8 | 2.0% | 9 |  | 9 | 3.4% | 18 | 1.9% |  |
| 12 |  | 14 | 14 | 5.1% |  |  |  | 0.0% |  |  |  | 0.0% | 14 | 1.5% |  |
| 13 | 3 |  | 3 | 1.1% | 26 |  | 26 | 6.5% | 12 |  | 12 | 4.5% | 41 | 4.4% |  |
| 14 | 2 |  | 2 | 0.7% | 10 |  | 10 | 2.5% | 7 |  | 7 | 2.6% | 19 | 2.0% |  |
| 15 | 1 |  | 1 | 0.4% | 14 |  | 14 | 3.5% | 4 |  | 4 | 1.5% | 19 | 2.0% |  |
| 16A | 2 |  | 2 | 0.7% | 7 |  | 7 | 1.8% | 2 |  | 2 | 0.8% | 11 | 1.2% |  |
| 16B | 2 |  | 2 | 0.7% | 6 |  | 6 | 1.5% | 5 |  | 5 | 1.9% | 13 | 1.4% |  |
| 17 |  | 18 | 18 | 6.6% |  |  |  | 0.0% |  |  |  | 0.0% | 18 | 1.9% |  |
| 18 | 9 |  | 9 | 3.3% | 23 |  | 23 | 5.8% | 1 |  | 1 | 0.4% | 33 | 3.5% |  |
| 19A | 5 |  | 5 | 1.8% | 8 |  | 8 | 2.0% | 2 |  | 2 | 0.8% | 15 | 1.6% |  |
| 19B | 1 |  | 1 | 0.4% | 12 |  | 12 | 3.0% | 8 |  | 8 | 3.0% | 21 | 2.2% |  |
| 20 | 10 |  | 10 | 3.6% | 22 |  | 22 | 5.5% | 12 |  | 12 | 4.5% | 44 | 4.7% |  |
| 21 | 4 | 5 | 9 | 3.3% | 10 | 1 | 11 | 2.8% | 8 |  | 8 | 3.0% | 28 | 3.0% |  |
| 22 |  | 14 | 14 | 5.1% | 1 |  | 1 | 0.3% | 12 |  | 12 | 4.5% | 27 | 2.9% |  |
| 23 |  | 15 | 15 | 5.5% | 10 | 1 | 11 | 2.8% | 15 |  | 15 | 5.7% | 41 | 4.4% |  |
| 24 |  |  |  | 0.0% | 18 |  | 18 | 4.5% | 17 |  | 17 | 6.4% | 35 | 3.7% |  |
| 25 | 2 |  | 2 | 0.7% | 14 |  | 14 | 3.5% | 16 |  | 16 | 6.0% | 32 | 3.4% |  |
| 26A | 12 |  | 12 | 4.4% | 15 |  | 15 | 3.8% | 10 |  | 10 | 3.8% | 37 | 3.9% |  |
| 26B | 10 |  | 10 | 3.6% | 26 |  | 26 | 6.5% | 11 |  | 11 | 4.2% | 47 | 5.0% |  |
| 27 | 19 | 20 | 39 | 14.2% | 35 | 2 | 37 | 9.3% | 18 | 1 | 19 | 7.2% | 95 | 10.1% |  |
| **Grand Total** | **160** | **114** | **274** | **100.0%** | **390** | **8** | **398** | **100.0%** | **261** | **4** | **265** | **100.0%** | **937** | **100.0%** |  |
