# Supplementary material for: Frequency-dependent selection can forecast evolution in Streptococcus pneumoniae
Source: PLoS Biol. 2020 Oct 22;18(10):e3000878. doi: 10.1371/journal.pbio.3000878 (PMC7580979; doi:10.1371/journal.pbio.3000878)
Supplement: S2 Table — For each figure, we have listed the analysis, dataset used (SW US or Mass), sample used, strains included, and explanation of any strain exclusions. Mass, Massachusetts; SW US, Southwest US. (DOCX) [file pbio.3000878.s007.docx]

| **Figure** | **Analysis** | **Dataset** | **Sample** | **Strains** | **Explanation** |
| --- | --- | --- | --- | --- | --- |
| 1A | Descriptive | SW US | Entire sample | 35/35 | pre-vaccine to post-vaccine change |
| 1B | Pro-rata model | SW US | Entire sample | 35/35 | pre-vaccine to post-vaccine change |
| 2 | Predicted fitness | Simulation | N/A | 35/35 | Predicted fitness simulation |
| 3A | Predicted fitness of all NVT strains | SW US | Entire sample | 31/35 | 3 strains are VT only, 1 strain is polyphyletic (n=4 excluded)  4 NVT strains were not observed pre-vaccine and were imputed from a later time point (n=4 imputed) |
| 3B | Quadratic programming of pre-vaccine NVT sample only | SW US | Pre-vaccine only | 27/35 | 3 strains are VT only, 1 strain is polyphyletic (n=4 excluded)  4 NVT strains were not observed pre-vaccine (n=4 excluded) |
| 3C | Quadratic programming model evaluation (Fig 3B) | SW US | Pre-vaccine only | 27/35 | 3 strains are VT only, 1 strain is polyphyletic (n=4 excluded)  4 NVT strains were not observed pre-vaccine (n=4 excluded) |
| 3D | Quadratic programming model evaluation (Fig 3B) | SW US | Pre-vaccine only | 27/35 | 3 strains are VT only, 1 strain is polyphyletic (n=4 excluded)  4 NVT strains were not observed pre-vaccine (n=4 excluded) |
| S1 | Phylogenetic analysis/ population structure | SW US | Entire sample | 937 genomes (35 strains) |  |
| S2 A | Descriptive | Mass | Entire sample | 16/16 | * peri-vaccine to post-vaccine change |
| S2 B | Pro-rata model | Mass | Entire sample | 16/16 | * peri-vaccine to post-vaccine change |
| S2 C | Quadratic programming of pre-vaccine NVT only | Mass | *Peri-vaccine only | 9/16 | 1 strain is VT only, 1 strain is polyphyletic (n=2 excluded)  5 NVT strains were not observed pre-vaccine (n=5 excluded) |
| S2 D | Quadratic programming model evaluation (Fig S2C) | Mass | *Peri-vaccine only | 9/16 | 1 strain is VT only, 1 strain is polyphyletic (n=2 excluded)  5 NVT strains were not observed pre-vaccine (n=5 excluded) |

* While the Southwest US dataset contains a true pre-vaccine, sample collected prior to the introduction of PCV7, the Massachusetts sample was collected after the PCV7 was introduced into the population. For this reason, we have termed the initial time point in the Massachusetts sample ‘peri-vaccine’.
