# Supplementary material for: Frequency-dependent selection can forecast evolution in Streptococcus pneumoniae
Source: PLoS Biol. 2020 Oct 22;18(10):e3000878. doi: 10.1371/journal.pbio.3000878 (PMC7580979; doi:10.1371/journal.pbio.3000878)
Supplement: S1 Code — Rmd and pdf files for all code used replicate each analysis and reproduce main and supporting figures. Source data is provided in supporting data. (ZIP) [file pbio.3000878.s008.zip › S1_Code.pdf]

Predicting evolution using frequency-dependent selection in bacterial populations

Data analysis and simulations

Taj Azarian and Pamela P. Martinez

Contents

Figure 1: Southwest US dataset 2
Figure 2: Simulations 4
Figure 3: Prediction 8
Figure S1: Phylogeny Southwest US dataset 17
Figure S2: Prediction Mass US dataset 24
Figure S3: Core versus Accessory Genome distances 32
Figure S4: Distribution of COGs by SCs 36
Table 1: Statistics 37

This is the code for reproducing the figures from the paper "Predicting evolution using frequency-dependent selection in bacterial populations" ## add DOI

```
require(ape)
require(car)
require(vegan)
require(readxl)
require(gtools)
require(cowplot)
require(deSolve)
require(ggrepel)
require(Metrics)
require(quadprog)
require(openxlsx)
require(tidyverse)
# require(ggtree)
# require(gridSVG)
# require(svglite)
# require(phytools)



#### Quadratic Programming function ####
## X is a matrix with rows = COGs and columns = SCs
## Y is a matrix with rows = COGs and columns = 1

QP <- function(X, Y){
  rinv <- solve(chol(t(X) %*% X)) # M to be minimized in quad. function (Choleski decomp)
  C <- cbind(rep(1,ncol(X)), diag(ncol(X))) #Contraints to minimize the quad. function
```

```
  b <- c(1,rep(0,ncol(X)))
  d <- t(Y) %*% X  #Vector in the quadratic function to be minimized
  output <- solve.QP(Dmat = rinv, factorized = TRUE, dvec = d,
                     Amat = C, bvec = b, meq = 1)$solution
  output <- round(output, digits = 5)
  return(output)
}
```

Figure 1: Southwest US dataset

```
#### Data mining ####
set.seed(9340)
df <- read_csv("data_southwestUS.csv") %>% rename(SC = BAPS2) ## metadata
dfpre <- df %>% subset(Epoch1 == "E1") ## E1 is the pre-vaccine data
vaccineT <- df %>% distinct(SC, PCV7.actual)
vaccineT <- vaccineT %>%
  subset(PCV7.actual == "VT") %>%
  rename(W = PCV7.actual) %>%
  full_join(subset(vaccineT,PCV7.actual == "NVT")) %>%
  unite("Vaccine", W:PCV7.actual, na.rm = TRUE) %>%
  mutate(Vaccine = recode_factor(Vaccine, NVT = "Non-vaccine type",
          VT = "Vaccine type",  VT_NVT = "Mixed type"))

dfFig1 <- df %>% select(SC, Epoch1) %>% group_by(Epoch1) %>%
  count(SC) %>% mutate(freq = prop.table(n)) %>% ungroup() %>%
  select(Epoch1, SC, freq) %>%
  spread(Epoch1, freq, fill = 0)

zero_pre <- dfpre %>% distinct(SC) %>%
  mutate(n = 0, freq = 0)

#### Replicates  - null expectation Pro rata ####
replicates <- 10000

if(file.exists("dfpre_NVT_all.csv")){
  dfpre_NVT_all <- read_csv("dfpre_NVT_all.csv")
  dfpre_NVT_all_it <- read_csv("dfpre_NVT_all_it.csv")
} else {
  dfpre_NVT_all_it <- data.frame(NULL)
  for(i in 1:replicates){
    #sub-sampling from each epoch independently with replacement
    dfpre_NVT_i <- dfpre %>% sample_frac(1, replace = TRUE) %>%
      subset(PCV7.actual == "NVT") %>% count(SC) %>%
      mutate(freq = prop.table(n)) %>% bind_rows(zero_pre) %>%
      group_by(SC) %>% summarise(n = sum(n), freq = sum(freq)) %>%
      arrange(SC) %>% ungroup %>% mutate(iter = i)

    dfpre_NVT_all_it <- bind_rows(dfpre_NVT_all_it, dfpre_NVT_i)
  }
  dfpre_NVT_all <- dfpre_NVT_all_it %>% group_by(SC) %>%
  summarise(expected_E3 = quantile(freq, 0.5),
```

```r
            cil = quantile(freq, 0.025),
            ciu = quantile(freq, 0.975)) %>%
  ungroup()
  write_csv(dfpre_NVT_all, "dfpre_NVT_all.csv")
  write_csv(dfpre_NVT_all_it, "dfpre_NVT_all_it.csv")
}

dfFig1 <- left_join(dfFig1, dfpre_NVT_all) %>%
  replace(., is.na(.), 0) %>% mutate(Delta = E3 - E1,
         DeltaExp = expected_E3 - E1,
         CI_low = cil-E1, CI_up = ciu-E1) %>%
  mutate(Signif = ifelse(Delta > CI_up, "+", NA)) %>%
  mutate(Signif = ifelse(Delta < CI_low, "-", Signif)) %>%
  mutate(Signif = ifelse(E1 == 0 | E3 == 0, NA, Signif)) %>%
  left_join(vaccineT)

#### Plot A: Prevalence by sequence cluster ####
datPlotA <- dfFig1 %>% select(SC, E1, E3) %>%
  pivot_longer(-SC, names_to =  "Epoch", values_to = "Prevalence") %>%
  left_join(vaccineT) %>% arrange(Epoch,-Prevalence) %>%
  mutate(Epoch = recode_factor(Epoch,
         E1 = "Pre-vaccine", E3 = "Post-vaccine"),
         SC = fct_relevel(SC, levels = unique(SC)))

plot1A <- ggplot(datPlotA, aes(x=SC, y=Prevalence, alpha=Epoch, fill=Vaccine)) +
  geom_bar(stat='identity', position='dodge') +
  scale_alpha_manual(values = c(1,0.4)) +
  scale_fill_manual(values = c("#004488","#BB5566","#DDAA33"),
                    name = "Composition") +
  xlab("Strain (SC)") + theme_classic() +
  scale_y_continuous(expand = c(0, 0), limits = c(0,0.16),
                     breaks=c(0,0.03,0.06,0.09,0.12,0.15)) +
  theme(legend.key.size = unit(0.6, "lines"),
        axis.title = element_text(size = 8),
        axis.text = element_text(size = 6, colour = "black"),
        legend.title = element_text(face="bold", size = 7),
        legend.text=element_text(size=6),
        legend.justification = c(1, 1), legend.box = "horizontal",
        legend.position = c(1, 1), # legend.position = c(0.725, 0.85),
        legend.spacing.y = unit(0.1, "cm"),
        legend.background = element_blank(),
        legend.box.background = element_rect(fill = gray(0.96), color = NA))

#### Plot B: Change in prevalence ####
datPlotB <- dfFig1 %>% select(SC, Delta:Vaccine) %>%
  mutate(SC = factor(SC, levels = levels(datPlotA$SC)))

plot1B <- datPlotB %>% ggplot() + theme_classic() +
  geom_hline(yintercept=0, lty="dashed",size=0.5) +
  geom_point(aes(SC, Delta, col = Vaccine),
             size=3, show.legend = F, alpha = 0.9) +
  geom_pointrange(aes(x=SC, y=DeltaExp,
             ymin=CI_low, ymax=CI_up),
```

```r
           size=.1, fatten = 3, show.legend = F) +
  geom_point(aes(x=SC, y=-0.095, shape = Signif), size=5,
           col = "lightcyan4", show.legend = F) +
  scale_colour_manual(values = c("#004488","#BB5566","#DDAA33")) +
  scale_shape_identity() +
  scale_y_continuous(limits = c(-0.1,0.08),
           breaks=c(-0.08,-0.04,0,0.04,0.08)) +
  labs(x="Strain (SC)") + labs(y="Prevalence Change") +
  theme(axis.title = element_text(size = 8),
        axis.text = element_text(size = 6, colour = "black"))

#### Final figure 1 files ####
figure1 <- plot_grid(plot1A, plot1B, labels = c("A","B"), ncol = 1, label_size = 10)
setwd("PLOS_final_version_figures")
ggsave("Fig1.png", figure1, width = 19, height = 19/2, units = "cm")
ggsave("Fig1.pdf", figure1, width = 19, height = 19/2, units = "cm")
ggsave("Fig1.tiff", figure1, width = 19, height = 19/2, units = "cm")

listFig1 <- list(figure_1_A= datPlotA, figure_1_B = datPlotB,
          figure_1_B_iter = dfpre_NVT_all_it)
openxlsx::write.xlsx(listFig1, file = "Fig1_data.xlsx")
```

Figure 2: Simulations

```r
#### Funct. rootfun ####
rfun <- function(t, state, pars){
  dstate <- unlist(repEq(t, state, pars)) # rate of change vector
  return(sum(abs(dstate)) - 1e-4)
}

#### Funct. checkFeas ####
checkFeas <- function(e, g){
  g <- as.data.frame(g)
  temp <- sapply(g, function(x)(length(unique(x))))
  id <- which(temp==1)
  e[id] <- as.numeric(unique(g[,id]))
  return(e)
}

#### Funct. replicator ####
repEq <- function(t, Nf, pars){
  with(as.list(c(Nf, pars)), {
    f <- NULL
    dfdt <- rep(0, nSC)
    xifi <- rep(0, nSC)

    ## loci frequencies ##
    for(k in 1:nCOG){ f[k] <- sum(Nf*genot[,k]) }

    for(k in 1:nSC){
      for(l in 1:nCOG){
```

```
        xifi[k] <- xifi[k] + Nf[k]*(genot[k,l]*(eqbm[l] - f[l]))
      }
    }

    ## dfdt ##
    for(k in 1:nSC){ dfdt[k] <- xifi[k] - Nf[k]*sum(xifi) }

    return(list(dfdt))
  })
}


####################################
#### Plot A ####

#### Pre-intervation ####
#### Parameters ####
nCOG <- 10
nSC <- 8
eqbm <- c(0.5677,0.5138,0.4050,0.4388,0.4981,
          0.5065,0.5725,0.4513,0.5811,0.4034)
vacT <- c(2,3,5)
timeSteps <- 0.5
posCom <- 2^nCOG
genot <- data.frame(permutations(n=2,r=nCOG,v=c(0,1), repeats.allowed = T))
colnames(genot) <- as.character(1:nCOG)
pres <- c(7,193,320,337,340,621,674,842)
genotF <- c(0.2342,0.1511,0.0033,0.1248, 0.1750,0.0067,0.2219,0.083)
genotP <- genot[pres,]
times <- seq(from=0, to=1000, by=timeSteps)
pars <- list(eqbm = eqbm, nCOG = nCOG, nSC = nSC, genot = genotP)

#### Simulations ####
out1 <- out1P <- as.data.frame(lsodar(func=repEq,y=genotF,
                    times=times,parms=pars,rootfun=rfun))
out1 <- out1 %>% pivot_longer(-time, names_to="genotype", values_to="frequency") %>%
  mutate(genotype = paste('G', genotype, sep = "")) %>% subset(frequency > 0)

out1_pre <- out1 %>% mutate(time = time/3)
E1 <- round(as.numeric(out1P[nrow(out1P),-1]), digits = 5) ### last time in the data frame

#### Intervention ####
#### Parameters ####
E2 <- E1
E2[vacT] <- 0 ### remove genotypes G2 (001) and G6 (101) 'vaccine types'
E2 <- E2/sum(E2)
idZ <- which(E2 > 0)
timeS <- 40
genotPV <- genotP[idZ,]
eqbmPV <- checkFeas(eqbm, genotPV)
pars$eqbm <- eqbmPV
pars <- list(eqbm = eqbm, nCOG = nCOG, nSC = nSC, genot = genotP)
```

```r
rfun <- function(t, state, pars){
  dstate <- unlist(repEq(t, state, pars)) # rate of change vector
  return(sum(abs(dstate)) - 1e-6)
}

### Simulations ####
out2 <- out2P <- as.data.frame(lsodar(func=repEq,y=E2,
                    times=times,parms=pars,rootfun=rfun))
out2 <- out2 %>% mutate(time = time + timeS) %>%
  pivot_longer(-time, names_to="genotype", values_to="frequency") %>%
  mutate(genotype = paste('G', genotype, sep = "")) %>% subset(time <= 80)

datPlotA <- rbind(out1_pre, out2)
E3 <- round(as.numeric(out2P[nrow(out2P),-1]), digits = 5)

lineT <- data.frame(linetype = "Non-vaccine type",
                    genotype = paste('G', 1:8, sep = ""), index = 1:8)
lineT <- lineT %>% mutate(linetype = ifelse(index %in% vacT,
                    "Vaccine type", "Non-vaccine type"), index = NULL)

datPlotA <- datPlotA %>% full_join(lineT) %>%
  mutate(frequency = ifelse(frequency == 0,
                     runif(1, min = 0.00001, max = 0.00002), frequency))

E3 <- datPlotA %>% subset(time == max(datPlotA$time)) %>% arrange(-frequency)
alphaT <- data.frame(genotype = E3$genotype,
                     alpha = c(seq(from=1,to=0.9,length.out =5),1,1,1))
datPlotA <- full_join(datPlotA, alphaT)


#### Figure A ####
plot2A <- datPlotA %>% ggplot() + theme_classic() +
  annotate("rect", xmin = 30.5, xmax = 37.5, ymin = -Inf,
           ymax = 0.28, fill = "gray93", colour = NA) +
  annotate("rect", xmin = 68.5, xmax = 75.5, ymin = -Inf,
           ymax = 0.28, fill = "gray93", colour = NA) +
  annotate("rect", xmin = 40, xmax = 42, ymin = -Inf, ymax = 0.28,
           fill = "darkslategray4", colour = NA, alpha = 0.3) +
  geom_line(aes(time, frequency, group = genotype,
                alpha = alpha, colour = factor(linetype),
                linetype = factor(linetype)), size = 0.7) +
  geom_segment(x = 38.2, y = 0.345, xend = 38.2, yend = 0.265,
               lineend = "butt", linejoin = "mitre", size = 0.4,
               arrow = arrow(length = unit(0.15, "cm")),
               colour = 'black', show.legend = F) +
  annotate("text", x = 38.2, y = 0.36, label="Vaccine introduction",
           fontface="bold", color="black", size = 3) +
  annotate("text", x = 33, y = 0.31, label="Pre-vaccine\nequilibrium",
           color="dimgrey", fontface="bold", size = 2.1) +
  annotate("text", x = 72, y = 0.31, label="Post-vaccine\nequilibrium",
           color="dimgrey", fontface="bold", size = 2.1) +
  annotate("text", x = 42.5, y = 0.31, label="Predicted\nFitness",
           color="darkslategray", fontface="bold", size = 2.1) +
```

```r
  scale_colour_manual(values = c('#004488', "#BB5566")) + xlim(0,76) +
  scale_y_continuous(breaks = c(0,0.1,0.2,0.3))+
  scale_alpha(range = c(0.15, 1), guide = 'none') +
  ylab("Prevalence") + xlab("\nTime") +
  theme(axis.title = element_text(size = 8),
        axis.text.y = element_text(size = 6, colour = "black"),
        axis.text.x = element_blank(),
        legend.spacing.y = unit(0, "pt"),
        legend.key.size = unit(0.6, "lines"),
        legend.title = element_blank(),
        legend.text=element_text(size=6),
        legend.position = c(0.125,0.9),
        legend.background = element_blank(),
        legend.box.background = element_rect(fill = gray(0.96), color = NA))


####################################
#### Plot B ####

SCs <- 35
COGs <- 2371 ## 2371
VTselect <- 3
replicates <- 10
datPlotB <- data.frame(NULL)

for(int in 1:replicates){
  el0 <- runif(COGs, min = 0.05, max = 0.95)
  dat <- as.matrix(replicate(SCs, sample(c(0,1), COGs, replace = TRUE)))
  dat <- unique(dat, MARGIN = 2)
  VT <- sample(1:SCs, VTselect)
  NVT <- (1:SCs)[-VT]

  if(ncol(dat) < SCs){
    cat("run again\n")
    break
  }

  #### E1 = pre-vaccine frequencies ####
  x1 <- QP(dat, as.matrix(el0))

  ### re-calculate el ###
  el <- dat %*% as.matrix(x1)

  #### E2 - frequencies just after vaccine intro ####
  x2 <- x1[-VT]
  x2 <- round(x2/sum(x2), digits = 5)
  dat2 <- dat[,-VT]
  fl <- dat2 %*% as.matrix(x2)

  #### E3 - frequencies long-term post-vaccine ####
  x3 <- QP(dat2, el)
  el3 <- dat2 %*% as.matrix(x3)
```

```r
  #### fitness function "omega" just after vaccine intro ####
  whole <- as.numeric(el - fl) ### this is similar to (el - fl) and thus fitness.
  deltaE <- x3 - x2
  omega <- as.vector(t(dat2) %*% whole) ## length SCs, similar to FFS

  phi <- sum(x2 * omega) ## average fitness
  rateOfChange <- omega - phi ## "rate of change": omega_g - phi

  ################### Relative fitness ###################
  dataOut <- data.frame(riskDif = deltaE, r = rateOfChange)
  dataOut <- subset(dataOut, riskDif != 0)
  dataOut <- mutate(dataOut, change =
              ifelse(riskDif < 0, "Decreased", "Increased"), rep = int)
  datPlotB <- rbind(datPlotB, dataOut)
}

datPlotB <- datPlotB %>% mutate(col =
              ifelse(sign(riskDif) == sign(r), "same", "diff"))

#### Figure B ####
plot2B <- datPlotB %>% ggplot() + theme_classic() +
  geom_point(aes(x=change, y=r, group=factor(rep),
                 colour = col), position = position_dodge(width = 1),
             size = 1.5, alpha  = 0.6) +
  geom_hline(yintercept = 0, linetype = 3) +
  ylab("Predicted Fitness") + xlab("Observed prevalence change") +
  scale_colour_manual(values = c("gray70", "darkslategray4")) +
  theme(legend.position="none",
        axis.title = element_text(size = 8),
        axis.text = element_text(size = 6, colour = "black"))

#### Final figure 2 ####
figure2 <- plot_grid(plot2A, plot2B, labels = c("A", "B"), rel_widths = c(1.75,1), label_size = 10)
setwd("PLOS_final_version_figures")
ggsave("Fig2.png", figure2, width = 19, height = 6, units = "cm")
ggsave("Fig2.pdf", figure2, width = 19, height = 6, units = "cm")
ggsave("Fig2.tiff", figure2, width = 19, height = 6, units = "cm")

listFig2 <- list(figure_2_A= datPlotA, figure_2_B = datPlotB)
openxlsx::write.xlsx(listFig2, file = "Fig2_data.xlsx")
```

Figure 3: Prediction

```r
#### Data ####
dfFVT <- df %>% select(SC,PCV7.actual, Epoch1) %>%
  group_by(Epoch1) %>%  count(SC,PCV7.actual) %>%
  mutate(freq = round(prop.table(n), digits = 3)) %>%
  ungroup() %>% select(Epoch1, SC, PCV7.actual, freq) %>%
  spread(Epoch1, freq, fill = 0) %>% arrange(SC,PCV7.actual)

#### Present at E1 ####
```

```r
SCE1 <- dfFVT %>% subset(E1 > 0) %>% ## & SC !="27"
  select(SC, PCV7.actual) %>%
  mutate(Epoch1 = "E1")

#### NVTs Present at E1 ####
SCE1NVT <- SCE1 %>% subset(PCV7.actual == "NVT" & SC !="27")

SC_freq_df <- df %>%
  select(SC, PCV7.actual, Epoch1, HMPREF0837_12128:HMPREF0837_10616) %>%
  arrange(SC) %>% group_by(SC,PCV7.actual,Epoch1) %>%
  mutate(SC_n = n()) %>% ungroup() %>%
  group_by(SC,PCV7.actual,Epoch1,SC_n) %>%
  summarise_at(vars(HMPREF0837_12128:HMPREF0837_10616), mean) %>%
  ungroup()

### Get the matrix and the SC for the pre-vaccine epch "E1"
df_preV <- SCE1 %>% left_join(SC_freq_df)
SC_freq_preV <- as.matrix(df_preV %>% mutate(SC_freq=SC_n/sum(SC_n)) %>% select(SC_freq))
SC_COG_preV <- as.matrix(t(df_preV %>% select(HMPREF0837_12128:HMPREF0837_10616)))

#### Get e_l ####
el <- SC_COG_preV %*% SC_freq_preV

#### Figure A ####
dfImputed <- dfFVT %>% subset(PCV7.actual == "NVT" & E1 == 0) %>%
  select(SC,PCV7.actual) %>% mutate(Epoch1 = "E1", n = 1) %>%
  select(Epoch1,SC,PCV7.actual,n)

dfFVTImputed <- df %>% select(SC,PCV7.actual, Epoch1) %>%
  group_by(Epoch1) %>% count(SC,PCV7.actual) %>%
  ungroup() %>% bind_rows(dfImputed) %>% group_by(Epoch1) %>%
  mutate(freq = round(prop.table(n), digits = 3)) %>% ungroup() %>%
  select(Epoch1, SC, PCV7.actual, freq) %>%
  spread(Epoch1, freq, fill = 0) %>% arrange(SC,PCV7.actual)

dfFNVTImputed <- dfFVTImputed %>%
  subset(PCV7.actual == "NVT" & SC !="27") %>%
  mutate(deltaE = E3 - E1) %>% arrange(SC)

#### E2 - frequencies just after vaccine intro ####
x_imputed <- dfFNVTImputed$E1
x_imputed <- as.matrix(round(x_imputed/sum(x_imputed), digits = 5))

dat2_imputed <- dfImputed %>% mutate(Epoch1 = "E2") %>%
  select(SC,PCV7.actual,Epoch1) %>% bind_rows(SCE1NVT) %>%
  arrange(SC) %>% left_join(SC_freq_df) %>%
  select(HMPREF0837_12128:HMPREF0837_10616)
dat2_imputed <- as.matrix(dat2_imputed)

fl_imp <- t(dat2_imputed) %*% x_imputed

#### fitness function "omega" just after vaccine intro ####
whole <- as.numeric(el - fl_imp) ### this is similar to (el - fl) and thus fitness.
```

```r
deltaE <- dfFNVTImputed$deltaE
omega <- as.vector(dat2_imputed %*% whole) ## length SCs, similar to FFS

phi <- sum(x_imputed * omega) ## average fitness
rateOfChange <- omega - phi ## "rate of change": omega_g - phi

datPlotA <- dfFNVTImputed %>% select(SC, deltaE) %>%
  mutate(r = rateOfChange) %>% subset(deltaE != 0) %>%
  mutate(change = ifelse(deltaE < 0, "Decreased", "Increased")) %>%
  mutate(col = ifelse(sign(deltaE) == sign(r), "same", "diff")) %>%
  left_join(vaccineT)

lm3 <- lm(r~deltaE,datPlotA)
summary(lm3)
```

```
##
## Call:
## lm(formula = r ~ deltaE, data = datPlotA)
##
## Residuals:
##      Min       1Q   Median       3Q      Max
## -12.7964  -1.8504  -0.1459   2.3969  10.6031
##
## Coefficients:
##             Estimate Std. Error t value Pr(>|t|)
## (Intercept)   0.4151     1.0220   0.406    0.688
## deltaE      182.1310    38.6050   4.718 5.55e-05 ***
## ---
## Signif. codes:  0 '***' 0.001 '**' 0.01 '*' 0.05 '.' 0.1 ' ' 1
##
## Residual standard error: 4.995 on 29 degrees of freedom
## Multiple R-squared:  0.4342, Adjusted R-squared:  0.4147
## F-statistic: 22.26 on 1 and 29 DF,  p-value: 5.555e-05
```

```r
#### Figure A ####
plot3A <- datPlotA %>%
  ggplot(aes(x=deltaE, y=r, colour = Vaccine)) +
  geom_hline(yintercept=0, lty="dotted", alpha=.6) +
  geom_vline(xintercept=0, lty="dotted", alpha=.6) +
  annotate("rect", xmin=-Inf,xmax=0,ymin=-Inf,ymax=0,
           fill="darkslategray4", alpha= 0.15) +
  annotate("rect", xmin=0,xmax=Inf,ymin=0,ymax=Inf,
           fill="darkslategray4", alpha= 0.15) +
  geom_smooth(aes(group = 1), size = 0.5,
              color="grey70", method=lm,
              formula = y~x,  show.legend=FALSE, se=FALSE) +
  geom_point(size = 1, alpha = 0.9) +
  scale_colour_manual(values = c("#004488","#DDAA33"),
                      labels = c("Non-vaccine", "Mixed"),
                    name = "Composition") +
  theme_classic() + xlab("Observed Prevalence Change") +
  ylab("Predicted Fitness") + xlim(-0.03, 0.065) + ylim(-12,20) +
  theme(legend.position = c(1,0.01),
      legend.justification = c(1,0.01),
```

```r
      legend.background = element_blank(),
      legend.box.background = element_rect(fill = "gray95", colour= NA),
      legend.title = element_text(size = 4.5),
      legend.text = element_text(size = 4),
      legend.key.size = unit(0.4, "lines"),
      axis.title = element_text(size = 5.5),
      axis.text = element_text(size = 5, colour = "black")) +
  geom_text(aes(x=deltaE, y=r, label = paste("SC", SC, sep="-")),
            data = filter(datPlotA, col == "diff"),
            check_overlap = TRUE,
                  nudge_x = c(-0.006, 0, 0.004),
                  nudge_y = 1.8,
                  size = 1.2, segment.color = NA,
                  show.legend = FALSE)

#### Figure B ####
df_postV <- SCE1NVT %>% left_join(SC_freq_df)
SC_COG_postV <- as.matrix(t(df_postV %>% select(HMPREF0837_12128:HMPREF0837_10616)))

SC_freq_postV_obs <- SCE1NVT %>% mutate(Epoch1 = "E3") %>%
  left_join(SC_freq_df) %>%
  mutate(SC_freq=SC_n/sum(SC_n, na.rm = T)) %>%
  select(SC, PCV7.actual, SC_freq) %>%
  mutate(SC_freq = replace_na(SC_freq, 0))

## Predict postV frequencies
SC_freq_postV_pred <- QP(SC_COG_postV, el) #Matrix: rows = COGs, columns = (SCs - VT)
datPlotB <- SC_freq_postV_obs %>%
  mutate(SC_pred = SC_freq_postV_pred) %>%
  left_join(vaccineT)

outlier3B <- datPlotB %>% mutate(diff = abs(SC_freq - SC_pred))
outlier3B <- outlier3B %>% filter(diff %in% boxplot(outlier3B$diff, plot = FALSE)$out)

W_model3B <- lm(data=datPlotB,SC_freq~SC_pred);
summary(W_model3B)
```

```
##
## Call:
## lm(formula = SC_freq ~ SC_pred, data = datPlotB)
##
## Residuals:
##       Min        1Q    Median        3Q       Max
## -0.059467 -0.014478 -0.002457  0.015118  0.053442
##
## Coefficients:
##             Estimate Std. Error t value Pr(>|t|)
## (Intercept) 0.012366   0.008616   1.435  0.16364
## SC_pred     0.666107   0.198686   3.353  0.00255 **
## ---
## Signif. codes:  0 '***' 0.001 '**' 0.01 '*' 0.05 '.' 0.1 ' ' 1
##
## Residual standard error: 0.02329 on 25 degrees of freedom
## Multiple R-squared:  0.3101, Adjusted R-squared:  0.2826
```

```
## F-statistic: 11.24 on 1 and 25 DF,  p-value: 0.002551
```

```r
confint(W_model3B)
```

```
##                    2.5 %     97.5 %
## (Intercept) -0.005380061 0.03011185
## SC_pred      0.256905970 1.07530899
```

```r
linearHypothesis(W_model3B, c("(Intercept) = 0", "SC_pred = 1"), test = "Chisq")
```

```
## Linear hypothesis test
##
## Hypothesis:
## (Intercept) = 0
## SC_pred = 1
##
## Model 1: restricted model
## Model 2: SC_freq ~ SC_pred
##
##   Res.Df      RSS Df Sum of Sq  Chisq Pr(>Chisq)
## 1     27 0.015093
## 2     25 0.013561  2 0.0015319 2.8241     0.2436
```

```r
linearHypothesis(W_model3B, c("(Intercept) = 0", "SC_pred = 1"))
```

```
## Linear hypothesis test
##
## Hypothesis:
## (Intercept) = 0
## SC_pred = 1
##
## Model 1: restricted model
## Model 2: SC_freq ~ SC_pred
##
##   Res.Df      RSS Df Sum of Sq     F Pr(>F)
## 1     27 0.015093
## 2     25 0.013561  2 0.0015319 1.412 0.2624
```

```r
plot3B <- datPlotB %>%
  ggplot(aes(x = SC_pred, y = SC_freq, colour = Vaccine)) +
  geom_segment(aes(x=0,xend=0.12,y=0,yend=0.12),
               color="black",alpha=.7,lwd=0.5,lty=3) +
  theme(legend.position = "none") + theme_classic() +
  geom_smooth(method='lm', color="#899DA4" ,
              formula=y~x, alpha=0.3, lwd=.6,
              fullrange=T, linetype="blank", show.legend=F) +
  annotate(geom = "text", x=0.11, y =0.116,
           label = "1:1 line", angle = 45, size = 1.5) +
  geom_point(size=1, alpha = 0.9) +  ##
  scale_x_continuous("Predicted Prevalence (NFDS)",
                     breaks = seq(0,0.12,0.04))+
  scale_y_continuous("Observed Prevalence",
                     breaks = seq(0,0.12,0.04)) +
  coord_fixed(ratio = 1, xlim=c(0,0.12), ylim=c(0,0.12)) +
  scale_colour_manual(values = c("#004488","#DDAA33")) +
  theme(legend.position = "none",
        axis.title = element_text(size = 5.5),
```

```r
      axis.text = element_text(size = 5, colour = "black")) +
  geom_text_repel(aes(label = paste("SC", SC, sep = "-")),
        data = outlier3B, size = 1.2, segment.color = NA,
        nudge_y = 0.006, nudge_x = 0)

#### Figure C ####

SC_freq_E1 <- df_preV %>%
  mutate(SC_freq_E1=SC_n/sum(SC_n)) %>%
  select(SC,PCV7.actual,SC_freq_E1)

datPlotCD <- datPlotB %>%
  left_join(SC_freq_E1) %>%
  mutate(diff_pred = SC_pred - SC_freq_E1,
         diff_obs =  SC_freq - SC_freq_E1) %>%
  left_join(vaccineT)

datPlotCD <- datPlotCD %>%
  mutate(diff = abs(diff_pred - diff_obs)) %>%
  arrange(diff)

stats <- summary(lm(datPlotCD$diff_pred~datPlotCD$diff_obs))
ars <- round(stats$adj.r.squared, digits = 3)
sseE <- round(sse(datPlotCD$diff_obs,
                  datPlotCD$diff_pred), digits = 3)
maeE <- round(mae(datPlotCD$diff_obs,
                  datPlotCD$diff_pred),digits = 3)##Mean Absolute Error
rmseE <- round(rmse(datPlotCD$diff_obs,
                    datPlotCD$diff_pred),digits = 3) ##Root Mean Squared Error

accNFDS <- data.frame(Model = "Accesory genome (NFDS)", nloci = length(el),
                      adj.r.squared = ars, SSE = sseE, RMSE = rmseE)


W_model3C <- lm(data=datPlotCD, diff_obs~diff_pred);
summary(W_model3C)
```

```
##
## Call:
## lm(formula = diff_obs ~ diff_pred, data = datPlotCD)
##
## Residuals:
##       Min        1Q    Median        3Q       Max
## -0.067643 -0.011921 -0.002054  0.011661  0.057236
##
## Coefficients:
##             Estimate Std. Error t value Pr(>|t|)
## (Intercept) 0.003684   0.006783   0.543   0.5919
## diff_pred   0.795065   0.273307   2.909   0.0075 **
## ---
## Signif. codes:  0 '***' 0.001 '**' 0.01 '*' 0.05 '.' 0.1 ' ' 1
##
## Residual standard error: 0.0243 on 25 degrees of freedom
## Multiple R-squared:  0.2529, Adjusted R-squared:  0.223
```

```
## F-statistic: 8.463 on 1 and 25 DF,  p-value: 0.007504
```

```r
confint(W_model3C)
```

```
##                   2.5 %     97.5 %
## (Intercept) -0.01028661 0.01765401
## diff_pred    0.23217925 1.35795061
```

```r
linearHypothesis(W_model3C, c("(Intercept) = 0", "diff_pred = 1"), test = "Chisq")
```

```
## Linear hypothesis test
##
## Hypothesis:
## (Intercept) = 0
## diff_pred = 1
##
## Model 1: restricted model
## Model 2: diff_obs ~ diff_pred
##
##   Res.Df      RSS Df  Sum of Sq  Chisq Pr(>Chisq)
## 1     27 0.015093
## 2     25 0.014761  2 0.00033197 0.5623     0.7549
```

```r
outlier3C <- datPlotCD %>%
  filter(diff %in% boxplot(datPlotCD$diff, plot = FALSE)$out)

#### Figure C ####
plot3C <- datPlotCD %>%
  ggplot(aes(x = diff_pred, y = diff_obs, colour = Vaccine)) +
  geom_segment(aes(x=-0.05,xend=0.1,y=-0.05,yend=0.1),
               color="black",alpha=.7,lwd=0.5,lty=3) +
  geom_smooth(method='lm', color="gray80" ,formula=y~x,
              alpha=0.3, lwd=.6, fullrange=T,
              linetype="blank", show.legend=F) +
  annotate(geom = "text", x=0.085, y=0.093,
           label = "1:1 line", angle = 45, size = 1.5) +
  geom_point(size=1, alpha = 0.9) + theme_classic() +
  scale_colour_manual(values = c("#004488","#DDAA33"),
                      labels = c("Non-vaccine type", "Mixed"),
                      name = "SC Composition") +
  theme(legend.position = "none",
       axis.title = element_text(size = 5.5),
       axis.text = element_text(size = 5, colour = "black")) +
  xlab("Predicted Prevalence Change (NFDS)")+
  ylab("Observed Prevalence Change") +
  coord_fixed(ratio = 1, xlim=c(-0.05,0.1), ylim=c(-0.05,0.1)) +
  annotate("text", x=-0.049, y=0.09, size=1.2, hjust = 0,
           label=paste("SSE = ", sseE, "\nRMSE = ",
                       rmseE, "\nAdj. R2 = ", ars)) +
  geom_text_repel(aes(label = paste("SC", SC, sep = "-")),
                  data = outlier3C, size = 1.2, segment.color = NA,
        nudge_y = 0.0075, nudge_x = 0)

#### Figure D ####
datPlotCD <- datPlotCD %>%
  mutate(prorata = SC_freq_E1/sum(SC_freq_E1)) %>%
```

```r
  mutate(diff_predPro = prorata - SC_freq_E1)

stats <- summary(lm(datPlotCD$diff_predPro~datPlotCD$diff_obs))
ars <- round(stats$adj.r.squared, digits = 3)
sseE <- round(sse(datPlotCD$diff_obs,
                  datPlotCD$diff_predPro), digits = 3)
maeE <- round(mae(datPlotCD$diff_obs,
                  datPlotCD$diff_predPro),digits = 3)##Mean Absolute Error
rmseE <- round(rmse(datPlotCD$diff_obs,
                    datPlotCD$diff_predPro),digits = 3) ##Root Mean Squared Error

accProrata <- data.frame(Model = "Accesory genome (Pro rata)", nloci = length(el),
                         adj.r.squared = ars, SSE = sseE, RMSE = rmseE)

W_model3D <- lm(data=datPlotCD,diff_obs~diff_predPro);
summary(W_model3D)
```

```
##
## Call:
## lm(formula = diff_obs ~ diff_predPro, data = datPlotCD)
##
## Residuals:
##       Min        1Q    Median        3Q       Max
## -0.038569 -0.021763  0.001313  0.014551  0.053397
##
## Coefficients:
##               Estimate Std. Error t value Pr(>|t|)
## (Intercept)   0.027719   0.009368   2.959  0.00666 **
## diff_predPro -0.541862   0.431660  -1.255  0.22098
## ---
## Signif. codes:  0 '***' 0.001 '**' 0.01 '*' 0.05 '.' 0.1 ' ' 1
##
## Residual standard error: 0.02727 on 25 degrees of freedom
## Multiple R-squared:  0.05929,    Adjusted R-squared:  0.02167
## F-statistic: 1.576 on 1 and 25 DF,  p-value: 0.221
```

```r
confint(W_model3D)
```

```
##                    2.5 %     97.5 %
## (Intercept)   0.008425814 0.04701286
## diff_predPro -1.430881941 0.34715809
```

```r
linearHypothesis(W_model3D, c("(Intercept) = 0", "diff_predPro = 1"), test = "Chisq")
```

```
## Linear hypothesis test
##
## Hypothesis:
## (Intercept) = 0
## diff_predPro = 1
##
## Model 1: restricted model
## Model 2: diff_obs ~ diff_predPro
##
##   Res.Df      RSS Df Sum of Sq  Chisq Pr(>Chisq)
## 1     27 0.028071
```

```
## 2     25 0.018586  2 0.0094854 12.759   0.001696 **
## ---
## Signif. codes:  0 '***' 0.001 '**' 0.01 '*' 0.05 '.' 0.1 ' ' 1
```

```
plot3D <- datPlotCD %>%
  ggplot(aes(x = diff_predPro, y = diff_obs, colour = Vaccine)) +
  geom_segment(aes(x=-0.05,xend=0.1,y=-0.05,yend=0.1),
               color="black",alpha=.7,lwd=0.5,lty=3) +
  geom_smooth(method='lm', color="gray80" ,formula=y~x,
              alpha=0.3, lwd=.6, fullrange=T,
              linetype="blank", show.legend=F) +
  annotate(geom = "text", x=0.085, y=0.093,
           label = "1:1 line", angle = 45, size = 1.5) +
  geom_point(size = 1, alpha = 0.9) + theme_classic() +
  scale_colour_manual(values = c("#004488","#DDAA33"),
                      labels = c("Non-vaccine type", "Mixed"),
                      name = "SC Composition") +
  theme(legend.position = "none",
       axis.title = element_text(size = 5.5),
       axis.text = element_text(size = 5, colour = "black")) +
  xlab("Predicted Prevalence Change (Pro rata)")+
  coord_fixed(ratio = 1, xlim=c(-0.05,0.1), ylim=c(-0.05,0.1)) +
  ylab("Observed Prevalence Change") +
  annotate("text", x=-0.049, y=0.09, size=1.2, hjust = 0,
           label=paste("SSE = ", sseE, "\nRMSE = ",
                       rmseE, "\nAdj. R2 = ", ars))

#### Combine figure 3 ####
ptitle1 <- ggplot() + theme_void() +
  annotate("rect", fill = "darkslategray4", alpha = 0.3,
           xmin = 0, xmax = 1, ymin = 0, ymax = 1)
ptitle1 <- ggdraw(ptitle1) +
  draw_label("Predicted Fitness", fontface='bold',
             colour = "darkslategrey", size = 8) ## ,

pA <- plot_grid(plot3A, labels = c("A"), label_size = 8)
pA <- plot_grid(ptitle1, pA, ncol = 1, scale = c(1,0.9), rel_heights=c(0.075, 1))


ptitle2 <- ggplot() + theme_void() +
  annotate("rect", fill = "gray93", xmin = 0, xmax = 1, ymin = 0, ymax = 1)
ptitle2 <- ggdraw(ptitle2) +
  draw_label("Post-vaccine Equilibrium Frequencies",
             fontface='bold', colour = "gray30", size = 8) ## ,
pBCD <- plot_grid(plot3B, plot3C, plot3D, ncol = 3, labels = c("B","C","D"), label_size = 8)
pBCD <- plot_grid(ptitle2, pBCD, ncol = 1, scale = c(1,0.9), rel_heights=c(0.075, 1))
plot3 <- plot_grid(pA, pBCD, rel_widths = c(1,3), rel_heights = c(1,1))

setwd("PLOS_final_version_figures")
ggsave("Fig3.pdf", plot3, width = 19, height = 5, units = "cm")
ggsave("Fig3.png", plot3, width = 19, height = 5, units = "cm")
ggsave("Fig3.tiff", plot3, width = 19, height = 5, units = "cm")

listFig3 <- list(figure_3_A=datPlotA, figure_3_B=datPlotB, figure_3_CD=datPlotCD)
```

```
openxlsx::write.xlsx(listFig3, file = "Fig3_data.xlsx")
```

Figure S1: Phylogeny Southwest US dataset

```
## The tree was modified after being produced (style purposes)

readMatrix<-function(heatmapData){
  if (is.matrix(heatmapData)) {
    x = data.frame(heatmapData)
  }
  else if (is.data.frame(heatmapData)) {
    x = heatmapData
  }
  else {
    x<-read.csv(heatmapData,row.names=1)
  }
  x
}
getLayout<-function(infoFile,infoCols,heatmapData,barData,doBlocks,
                    treeWidth=10,infoWidth=10,dataWidth=30,edgeWidth=1,
                    labelHeight=10,mainHeight=100,barDataWidth=10,
                    blockPlotWidth=10) {
  # m = layout matrix
  # w = layout widths vector
  # h = layout height vector

  # tree
  w = c(edgeWidth,treeWidth)
  m<-cbind(c(0,0,0),c(0,1,0)) # first two columns, edge + tree
  x = 1

  # info
  if (!is.null(infoFile)) { # info is provided

    printCols = TRUE
    if (!is.null(infoCols)) {
      if (is.na(infoCols)) {
        printCols = FALSE
      }}

    if (printCols) {
      x = x + 1
      m<-cbind(m,c(0,x,0))
      w = c(w,infoWidth)
    }
  }

  # heatmap
  if (!is.null(heatmapData)) {
    x = x + 1
    m<-cbind(m,c(x+1,x,0)) # add heatmap & labels
```

```r
    x = x + 2
    m[1,2] = x # add heatmap scale above tree
    w = c(w,dataWidth)
  }

  # barplot
  if (!is.null(barData)) {
    x = x + 1
    m<-cbind(m,c(0,x,x+1)) # barplot and scale bar
    x = x + 1
    w = c(w,barDataWidth)
  }

  if (doBlocks) {
    x = x + 1
    m<-cbind(m,c(0,x,0)) # recomb blocks
    w = c(w,blockPlotWidth)
  }

  # empty edge column
  m<-cbind(m,c(0,0,0))
  w = c(w,edgeWidth)

  if(!is.null(heatmapData) | !is.null(barData)){h = c(labelHeight,mainHeight,labelHeight)}
  else{ h = c(edgeWidth,mainHeight,edgeWidth)}

  return(list(m=as.matrix(m),w=w,h=h))
}

plotTree<-function(tree,ladderise=NULL,heatmapData=NULL,barData=NULL,infoFile=NULL,
                   blockFile=NULL,snpFile=NULL,gapChar="?",genome_size=5E6,blwd=5,
                   block_colour="black",snp_colour="red",genome_offset=0,
                   colourNodesBy=NULL,infoCols=NULL,outputPDF=NULL,outputPNG=NULL,
                   w,h,heatmap.colours=rev(gray(seq(0,1,0.1))),tip.labels=F,
                   tipLabelSize=1,offset=0,tip.colour.cex=0.5,legend=T,
                   legend.pos="bottomleft",ancestral.reconstruction=F,cluster=NULL,
                   tipColours=NULL,lwd=1.5,axis=F,axisPos=3,edge.color="black",
                   infoCex=0.8,colLabelCex=0.8,treeWidth=10,infoWidth=10,dataWidth=30,
                   edgeWidth=1,labelHeight=10,mainHeight=100,barDataWidth=10,
                   blockPlotWidth=10,barDataCol=2,heatmapBreaks=NULL,
                   heatmapDecimalPlaces=1,vlines.heatmap=NULL,vlines.heatmap.col=2,
                   heatmap.blocks=NULL,pie.cex=0.5) {

  require(ape)

  # PREPARE TREE, CHOOSE LADDERISATION OR NOT, AND GET TIP ORDER
  if (is.character(tree)){
    t<-read.tree(tree)
  }
  else t<-tree
  if (is.null(ladderise))
  {
    tl<-t
```

```
}
else if (ladderise=="descending")
{
  tl<-ladderize(t, T)
}
else if (ladderise=="ascending")
{
  tl<-ladderize(t, F)
}
else if (!is.null(ladderise))
{
  print("Ladderise option should be exactly 'ascending' or 'descending'.
        Any other command will raise this error.
        Leave option empty to order branches as per input tree.")
}
tips<-tl$edge[,2]
tip.order<-tips[tips<=length(tl$tip.label)]
# for ordering data. note that for tiplabel(), the order is the same as in t$tip (= tl$tip)
tip.label.order<-tl$tip.label[tip.order]


# PREPARE HEATMAP DATA
if (!is.null(heatmapData)) {

  # read heatmap data and convert to data frame
  x<-readMatrix(heatmapData)

  # order rows of heatmap matrix to match tree
  y.ordered<-x[tip.label.order,]

  # reorder columns?
  if (!is.null(cluster)) {
    if (!(cluster==FALSE)) {

      if (cluster=="square" & ncol(y.ordered)==nrow(y.ordered)) {
        # order columns to match row order
        original_order<-1:nrow(x)
        names(original_order)<-rownames(x)
        reordered<-original_order[tip.label.order]
        y.ordered<-y.ordered[,rev(as.numeric(reordered))]
      }

      else {
        # cluster columns
        if (cluster==TRUE) {cluster="ward"} # set default clustering algorithm
        h<-hclust(dist(t(na.omit(y.ordered))),cluster)
        y.ordered<-y.ordered[,h$order]
      }

    }} # finished reordering columns

} # finished setting up heatmap data
```

```r
# PREPARE BAR PLOT
if (!is.null(barData)) {
  b<-readMatrix(barData)
  barData<-b[,1]
  names(barData)<-rownames(b)
}

# PREPARE INFO TO PRINT
if (!is.null(infoFile)) {
  info<-readMatrix(infoFile)
  info.ordered<-info[rev(tip.label.order),]
}
else {info.ordered=NULL}


# PREPARE DISCRETE TRAIT FOR COLOURING NODES AND INFERRING ANCESTRAL STATES
ancestral=NULL
nodeColourSuccess=NULL
if (!is.null(colourNodesBy) & !is.null(infoFile)) {

  if (colourNodesBy %in% colnames(info.ordered)) {
    nodeColourSuccess = TRUE
    loc1<-info.ordered[,which(colnames(info.ordered)==colourNodesBy)]

    # assign values
    tipLabelSet <- character(length(loc1))
    names(tipLabelSet) <- rownames(info.ordered)
    groups<-table(loc1,exclude="")
    n<-length(groups)
    groupNames<-names(groups)

    # set colours
    if (is.null(tipColours)){ colours<-rainbow(n) }
    else{ colours<-tipColours }

    # assign colours based on values
    for (i in 1:n) {
      g<-groupNames[i]
      tipLabelSet[loc1==g]<-colours[i]
    }
    tipLabelSet <- tipLabelSet[tl$tip]

    # ancestral reconstruction
    if (ancestral.reconstruction) { ancestral<-ace(loc1,tl,type="discrete") }

  }}
# finished with trait labels and ancestral reconstruction


# OPEN EXTERNAL DEVICE FOR DRAWING
# open PDF for drawing
if (!is.null(outputPDF)) {
  pdf(width=w,height=h,file=outputPDF)
```

```r
}
# open PNG for drawing
if (!is.null(outputPNG)) {
  png(width=w,height=h,file=outputPNG)
}


# SET UP LAYOUT FOR PLOTTING
doBlocks <- (!is.null(blockFile) | !is.null(snpFile))
l <- getLayout(infoFile,infoCols,heatmapData,barData,doBlocks,
               treeWidth=treeWidth,infoWidth=infoWidth,dataWidth=dataWidth,
               edgeWidth=edgeWidth,labelHeight=labelHeight,mainHeight=mainHeight,
               barDataWidth=barDataWidth,blockPlotWidth=blockPlotWidth)
layout(l$m, widths=l$w, heights=l$h)


# PLOT TREE
par(mar=rep(0,4))
tlp<-plot.phylo(tl,no.margin=T,show.tip.label=tip.labels,label.offset=offset,
                edge.width=lwd,edge.color=edge.color,xaxs="i", yaxs="i",
                y.lim=c(0.5,length(tl$tip)+0.5),cex=tipLabelSize)

# colour by trait
if (!is.null(nodeColourSuccess)) {
  tiplabels(col= tipLabelSet,pch=16,cex=tip.colour.cex)
  if (ancestral.reconstruction) { nodelabels(pie=ancestral$lik.anc,
                                             cex=pie.cex, piecol=colours) }
  if (legend) { legend(legend.pos,legend=groupNames,fill=colours) }
}

if (axis) { axisPhylo(axisPos) }

# PLOT INFO
if (!is.null(infoFile)) { # info is provided

  printCols = TRUE
  if (!is.null(infoCols)) {
    if (is.na(infoCols)) {
      printCols = FALSE
    }}

  if (printCols) {

    par(mar=rep(0,4))

    if(!is.null(infoCols)){infoColNumbers = which(colnames(info.ordered) %in% infoCols)}
    else { infoColNumbers = 1:ncol(info.ordered)}

    plot(NA,axes=F,pch="",xlim=c(0,length(infoColNumbers)+1.5),
         ylim=c(0.5,length(tl$tip)+0.5),xaxs="i",yaxs="i")

    # plot all info columns
    for (i in 1:length(infoColNumbers)) {
```

```
      j<-infoColNumbers[i]
      text(x=rep(i+1,nrow(info.ordered)+1),y=c((nrow(info.ordered)):1),
           info.ordered[,j],cex=infoCex)
    }
  }
}


# PLOT HEATMAP
if (!is.null(heatmapData)) {

  if (is.null(heatmapBreaks)){
    heatmapBreaks = seq(min(y.ordered,na.rm=T),max(y.ordered,na.rm=T),
                        length.out=length(heatmap.colours)+1)}

  # plot heatmap
  par(mar=rep(0,4), xpd=TRUE)
  image((1:ncol(y.ordered))-0.5,(1:nrow(y.ordered))-0.5,
        as.matrix(t(y.ordered)),
        col=heatmap.colours,breaks=heatmapBreaks,
        axes=F,xaxs="i", yaxs="i", xlab="",ylab="")

  # draw vertical lines over heatmap
  if (!is.null(vlines.heatmap)) {
    for (v in vlines.heatmap) {abline(v=v, col=vlines.heatmap.col)}
  }

  # overlay blocks on heatmap
  if (!is.null(heatmap.blocks)) {
    for(coords in heatmap.blocks){
      rect(xleft=coords[1], 0, coords[2],
           ncol(y.ordered), col=vlines.heatmap.col, border=NA)}
  }


  # data labels for heatmap
  par(mar=rep(0,4))
  plot(NA, axes=F, xaxs="i", yaxs="i", ylim=c(0,2), xlim=c(0.5,ncol(y.ordered)+0.5))
  text(1:ncol(y.ordered)-0.5,rep(0,ncol(x)),colnames(y.ordered),
       srt=90, cex=colLabelCex, pos=4)

  # scale for heatmap
  par(mar=c(2,0,0,2))
  image(as.matrix(seq(min(y.ordered,na.rm=T),max(y.ordered,na.rm=T),
                      length.out=length(heatmap.colours)+1)),
        col=heatmap.colours,yaxt="n",breaks=heatmapBreaks,axes=F)
  axis(1,at=heatmapBreaks[-length(heatmapBreaks)]/max(y.ordered,na.rm=T),
       labels=round(heatmapBreaks[-length(heatmapBreaks)],heatmapDecimalPlaces))
}

# BARPLOT
if (!is.null(barData)) {
  par(mar=rep(0,4))
```

```r
  barplot(barData[tip.label.order], horiz=T, axes=F, xaxs="i",
          yaxs="i", xlab="", ylab="", ylim=c(0.25,length(barData)+0.25),
          xlim=c((-1)*max(barData,na.rm=T)/20,max(barData,na.rm=T)),
          col=barDataCol,border=0,width=0.5,space=1,names.arg=NA)

  # scale for barData plot
  par(mar=c(2,0,0,0))
  plot(NA, yaxt="n", xaxs="i", yaxs="i", xlab="", ylab="", ylim=c(0,2),
       xlim=c((-1)*max(barData,na.rm=T)/20,max(barData,na.rm=T)),frame.plot=F)
}

# SNPS AND RECOMBINATION BLOCKS
if (doBlocks) {
  par(mar=rep(0,4))
  plot(NA,axes=F,pch="",xlim=c(genome_offset,genome_offset+genome_size+1.5),
       ylim=c(0.5,length(tl$tip)+0.5),xaxs="i",yaxs="i") # blank plotting area

  # plot snps
  if (!is.null(snpFile)) {
    # in case colnames start with numbers or
    # contain dashes, which R does not like as column headers
    snps<-read.csv(snpFile,header=F,row.names=1)
    snps_strainCols <- snps[1,] # column names = strain names
    snps<-snps[-1,] # drop strain names

    for (strain in tip.label.order){
      # print SNPs compared to ancestral alleles in column 1
      s <- rownames(snps)[(as.character(snps[,1]) !=
              as.character(snps[,which(snps_strainCols==strain)])) &
              (as.character(snps[,which(snps_strainCols==strain)])!=gapChar) &
              (as.character(snps[,1])!=gapChar)]
      y <- which(tip.label.order==strain)
      if (length(s)>0) {
        for (x in s) {
          points(x,y,pch="|",col=snp_colour,cex=0.25)
        }
      }
    }
  }

  # plot blocks
  if (!is.null(blockFile)){
    blocks<-read.delim(blockFile,header=F)
    for (i in 1:nrow(blocks)) {
      if (as.character(blocks[i,1]) %in% tip.label.order) {
        y <- which(tip.label.order==as.character(blocks[i,1]))
        x1 <- blocks[i,2]
        x2 <- blocks[i,3]
        lines(c(x1,x2),c(y,y),lwd=blwd,lend=2,col=block_colour)
      }
    }
  }
```

```r
  } # finished with SNPs and recomb blocks

  # CLOSE EXTERNAL DRAWING DEVICE
  if (!is.null(outputPDF) | !is.null(outputPNG)) {
    dev.off()
  }

  # RETURN ordered info and ancestral reconstruction object
  if (!is.null(heatmapData)){mat=as.matrix(t(y.ordered))}
  else {mat=NULL}
  return(list(info=info.ordered,anc=ancestral,mat=mat,strain_order=tip.label.order))
}


tree<-read.tree("RAxML_bestTree.All.Core.tre") #Core genome tree

heatmap_colors <- c("#ECCBAE", "#A42820", "#5F5647", "#9A8822", "#74A089", "#D8B70A",
                    "#046C9A", "#3F5151", "#4E2A1E", "#F2300F", "#FF0000", "#02401B",
                    "#D69C4E", "#FAD510", "#CB2314", "#273046", "#00A08A", "#A2A475",
                    "#ABDDDE", "#550307", "#354823", "#F5CDB4", "#F2AD00", "#972D15",
                    "#000000", "#E1BD6D", "#EABE94", "#F8AFA8", "#F98400", "#F1BB7B",
                    "#81A88D", "#0B775E", "#35274A", "#FDDDA0", "#f0f0f0", "#377eb8",
                    "#e41a1c","#899DA4", "#D67236")

plotTree(tree, heatmapData="NWMA_metadata_numerated_revised.csv",
         heatmap.colours=heatmap_colors, legend=T, tip.labels = FALSE,
         lwd=.85, treeWidth=10, dataWidth=3)
```

Figure S2: Prediction Mass US dataset

```r
dfMA <- read_csv("data_MassUS.csv")
dfpreMA <- dfMA %>% subset(Epoch1 == "E1") ## E1 prevaccine

vaccineMA <- dfMA %>% distinct(SC, PCV7.actual)
vaccineMA <- vaccineMA %>%
  subset(PCV7.actual == "VT") %>%
  rename(W = PCV7.actual) %>%
  full_join(subset(vaccineMA, PCV7.actual == "NVT")) %>%
  unite("Vaccine", W:PCV7.actual, na.rm = TRUE) %>%
  mutate(Vaccine = recode_factor(Vaccine, NVT = "Non-vaccine type",
            VT = "Vaccine type",  VT_NVT = "Mixed type"))

dfFMA <- dfMA %>% select(SC, Epoch1) %>% group_by(Epoch1) %>%
  count(SC) %>% mutate(freq = prop.table(n)) %>% ungroup() %>%
  select(Epoch1, SC, freq) %>% spread(Epoch1, freq, fill = 0)

zero_preMA <- dfpreMA %>% distinct(SC) %>%
  mutate(n = 0, freq = 0)

#### Replicates  - null expectation Pro rata ####
replicates <- 10000
```

```
if(file.exists("dfpreMA_NVT_all.csv")){
  dfpreMA_NVT_all <- read_csv("dfpreMA_NVT_all.csv")
  dfpreMA_NVT_all_it <- read_csv("dfpreMA_NVT_all_it.csv")
} else {
  dfpreMA_NVT_all_it <- data.frame(NULL)
  for(i in 1:replicates){
    #sub-sampling from each epoch independently with replacement
    dfpreMA_NVT_i <- dfpreMA %>% sample_frac(1, replace = TRUE) %>%
      subset(PCV7.actual == "NVT") %>% count(SC) %>%
      mutate(freq = prop.table(n)) %>% bind_rows(zero_preMA) %>%
      group_by(SC) %>% summarise(n = sum(n), freq = sum(freq)) %>%
      arrange(SC) %>% ungroup %>% mutate(iter = i)

    dfpreMA_NVT_all_it <- bind_rows(dfpreMA_NVT_all_it, dfpreMA_NVT_i)
  }
  dfpreMA_NVT_all <- dfpreMA_NVT_all_it %>% group_by(SC) %>%
  summarise(expected_E3 = quantile(freq, 0.5),
            cil = quantile(freq, 0.025),
            ciu = quantile(freq, 0.975)) %>%
  ungroup()
  write_csv(dfpreMA_NVT_all, "dfpreMA_NVT_all.csv")
  write_csv(dfpreMA_NVT_all_it, "dfpreMA_NVT_all_it.csv")
}

dfFMA <- left_join(dfFMA, dfpreMA_NVT_all) %>%
  replace(., is.na(.), 0) %>%
  mutate(Delta = E3 - E1, DeltaExp = expected_E3-E1,
         CI_low = cil-E1, CI_up = ciu-E1) %>%
  mutate(signif = ifelse(Delta > CI_up, "+", NA)) %>%
  mutate(signif = ifelse(Delta < CI_low, "-", signif)) %>%
  mutate(signif = ifelse(E1 == 0 | E3 == 0, NA, signif)) %>%
  left_join(vaccineMA)

#### Plot A: Prevalence by sequence cluster ####
datPlotA <- dfFMA %>% select(SC, E1, E3) %>%
  pivot_longer(-SC, names_to =  "Epoch", values_to = "Prevalence") %>%
  left_join(vaccineMA) %>% arrange(Epoch,-Prevalence) %>%
  mutate(SC = as.character(SC) , Epoch = recode_factor(Epoch,
         E1 = "Peri-vaccine - E1", E3 = "Peri-vaccine - E3"),
         SC = fct_relevel(SC, levels = unique(SC)))

plotSI2A <- datPlotA %>%
  ggplot(aes(x=SC, y=Prevalence, alpha=Epoch, fill=Vaccine)) +
  geom_bar(stat='identity', position='dodge') +
  scale_alpha_manual(values = c(1,0.4)) +
  scale_fill_manual(values = c("#004488","#BB5566","#DDAA33"),
        name = "Composition") + xlab("Strain (SC)") +
  theme_classic() + scale_y_continuous(expand = c(0, 0),
        limits = c(0,0.3), breaks = seq(0,0.25,0.05)) +
  theme(legend.key.size = unit(0.6, "lines"),
        axis.title = element_text(size = 8),
        axis.text = element_text(size = 6, colour = "black"),
        legend.title = element_text(face="bold", size = 7),
```

```
        legend.text=element_text(size=6),
        legend.justification = c(1, 1), legend.box = "horizontal",
        legend.position = c(1, 1), # legend.position = c(0.725, 0.85),
        legend.spacing.y = unit(0.1, "cm"),
        legend.background = element_blank(),
        legend.box.background = element_rect(fill = gray(0.96), color = NA))


#### Plot B: Change in prevalence ####
datPlotB <- dfFMA %>% select(SC, Delta:Vaccine) %>%
  mutate(SC = factor(SC, levels = levels(datPlotA$SC)))

plotSI2B <- datPlotB %>% ggplot() + theme_classic() +
  geom_hline(yintercept=0, lty="dashed",size=0.5) +
  geom_point(aes(SC, Delta, col = Vaccine), size = 2.5,
             alpha  = 0.9, show.legend = F) +
  geom_pointrange(aes(x=SC, y=DeltaExp, ymin=CI_low, ymax=CI_up),
                  size=.1, fatten = 3, show.legend = F) +
  geom_point(aes(x=SC, y=-0.2, shape = signif), size=4,
             col = "lightcyan4", show.legend = F) +
  scale_colour_manual(values = c("#004488","#BB5566","#DDAA33")) +
  scale_shape_identity() +
  scale_y_continuous(limits = c(-0.2,0.3),
             breaks = seq(-0.2,0.3,0.1)) +
  labs(x="Strain (SC)") + labs(y="Prevalence Change") +
  theme(axis.title = element_text(size = 8),
        axis.text = element_text(size = 6, colour = "black"))

####################
#### Plot C ####

dfFVTMA <- dfMA %>% select(SC,PCV7.actual, Epoch1) %>%
  group_by(Epoch1) %>%  count(SC,PCV7.actual) %>%
  mutate(freq = round(prop.table(n), digits = 3)) %>%
  ungroup() %>% select(Epoch1, SC, PCV7.actual, freq) %>%
  spread(Epoch1, freq, fill = 0) %>% arrange(SC,PCV7.actual)

#### Present at E1 (17 SCs)####
SCE1MA <- dfFVTMA %>% subset(E1 > 0) %>% ##
  select(SC, PCV7.actual) %>% mutate(Epoch1 = "E1")

#### NVT present at E1 (9 SCs)####
SCE1MANVT <- SCE1MA %>% subset(PCV7.actual == "NVT")

SC_freq_dfMA <- dfMA %>%
  select(SC,PCV7.actual,Epoch1,pbp1a:CLS343169) %>%
  arrange(SC) %>% group_by(SC,PCV7.actual,Epoch1) %>%
  mutate(SC_n = n()) %>% ungroup() %>%
  group_by(SC,PCV7.actual,Epoch1,SC_n) %>%
  summarise_at(vars(pbp1a:CLS343169),mean) %>%
  ungroup()

### Get the matrix and the SC for the pre-vaccine epch "E1"
```

```r
df_preVMA <- SCE1MA %>% left_join(SC_freq_dfMA)
SC_freq_preVMA <- as.matrix(df_preVMA %>%
                              mutate(SC_freq=SC_n/sum(SC_n)) %>% select(SC_freq))
SC_COG_preVMA <- as.matrix(t(df_preVMA %>% select(pbp1a:CLS343169)))

## Get e_l for the Mass data (1056 COGs)
el_MA <- SC_COG_preVMA %*% SC_freq_preVMA

#################################

df_postVMA <- SCE1MANVT %>% left_join(SC_freq_dfMA)
SC_COG_postVMA <- as.matrix(t(df_postVMA %>% select(pbp1a:CLS343169)))

#### Predict postV frequencies ####
SC_freq_postV_predMA <- QP(SC_COG_postVMA, el_MA) #Matrix: rows=COGs, cols=(SCs - VT)

SC_freq_postV_obsMA <- SCE1MANVT %>% mutate(Epoch1 = "E3") %>%
  left_join(SC_freq_dfMA) %>%
  mutate(SC_freq=SC_n/sum(SC_n, na.rm = T)) %>%
  select(SC, PCV7.actual, SC_freq) %>%
  mutate(SC_freq = replace_na(SC_freq, 0)) %>%
  mutate(SC_pred = SC_freq_postV_predMA) %>%
  left_join(vaccineMA)


datPlotC <- SC_freq_postV_obsMA %>% arrange(SC) %>%
  select(SC, Vaccine, SC_pred, SC_freq)

W_modelSC <- lm(data=datPlotC,SC_freq~SC_pred);
summary(W_modelSC)
```

```
##
## Call:
## lm(formula = SC_freq ~ SC_pred, data = datPlotC)
##
## Residuals:
##       Min        1Q    Median        3Q       Max
## -0.073407 -0.018085 -0.008067  0.027381  0.068476
##
## Coefficients:
##             Estimate Std. Error t value Pr(>|t|)
## (Intercept)  0.02328    0.02959   0.787   0.4572
## SC_pred      0.79044    0.22626   3.494   0.0101 *
## ---
## Signif. codes:  0 '***' 0.001 '**' 0.01 '*' 0.05 '.' 0.1 ' ' 1
##
## Residual standard error: 0.04684 on 7 degrees of freedom
## Multiple R-squared:  0.6355, Adjusted R-squared:  0.5834
## F-statistic:  12.2 on 1 and 7 DF,  p-value: 0.01008
```

```r
confint(W_modelSC)
```

```
##                   2.5 %     97.5 %
## (Intercept) -0.04669562 0.09326407
```

```
## SC_pred      0.25542617 1.32545776
```

```r
linearHypothesis(W_modelSC, c("(Intercept) = 0", "SC_pred = 1"), test = "Chisq")
```

```
## Linear hypothesis test
##
## Hypothesis:
## (Intercept) = 0
## SC_pred = 1
##
## Model 1: restricted model
## Model 2: SC_freq ~ SC_pred
##
##   Res.Df      RSS Df Sum of Sq  Chisq Pr(>Chisq)
## 1      9 0.017243
## 2      7 0.015361  2 0.0018824 0.8578     0.6512
```

```r
## no outliers
outlierSC <- datPlotC %>% mutate(diff = abs(SC_freq - SC_pred))
outlierSC <- outlierSC %>%
  filter(diff %in% boxplot(outlierSC$diff, plot = FALSE)$out)

#### Figure C ####
plotSI2C <- datPlotC %>%
  ggplot(aes(x = SC_pred, y = SC_freq, colour = Vaccine)) +
  theme_classic() +
  geom_segment(aes(x=0,xend=0.3,y=0,yend=0.3),
               color="black",alpha=.7,lwd=0.5,lty=3) +
  geom_smooth(method='lm',color="#899DA4",
              formula=y~x, alpha=0.3, lwd=.6,
              fullrange=T, linetype="blank", show.legend=F) +
  annotate(geom = "text", x=0.27, y =0.29,
           label = "1:1 line", angle = 45, size = 2) +
  geom_point(size = 2, alpha = 0.8) +
  scale_x_continuous("Predicted Prevalence (NFDS)",
                     breaks = seq(0,0.3,0.06))+
  scale_y_continuous("Observed Prevalence",
                     breaks = seq(0,0.3,0.06)) +
  coord_fixed(ratio = 1, ylim=c(0,0.3), xlim=c(0,0.3)) +
  scale_colour_manual(values = c("#004488","#DDAA33"),
                      labels = c("Non-vaccine", "Mixed"),
                    name = "Composition") +
  theme(legend.position = "none",
        axis.title = element_text(size = 8),
        axis.text = element_text(size = 6, colour = "black"))

####################################

SC_freq_E1MA <- df_preVMA %>%
  mutate(SC_freq_E1=SC_n/sum(SC_n)) %>%
  select(SC,PCV7.actual,SC_freq_E1)

datPlotDE <- SC_freq_postV_obsMA %>%
  left_join(SC_freq_E1MA) %>%
  mutate(diff_pred =  SC_pred - SC_freq_E1,
```

```r
         diff_obs = SC_freq - SC_freq_E1) %>%
  left_join(vaccineMA)  %>%
  mutate(diff = abs(diff_pred - diff_obs)) %>%
  arrange(diff)

stats <- summary(lm(datPlotDE$diff_pred~datPlotDE$diff_obs))
ars <- round(stats$adj.r.squared, digits = 3)
sseE <- round(sse(datPlotDE$diff_obs,
                  datPlotDE$diff_pred), digits = 3)
maeE <- round(mae(datPlotDE$diff_obs,
                  datPlotDE$diff_pred),digits = 3)##Mean Absolute Error
rmseE <- round(rmse(datPlotDE$diff_obs,
                    datPlotDE$diff_pred),digits = 3) ##Root Mean Squared Error

W_modelSD <- lm(data=datPlotDE,diff_obs~diff_pred);
summary(W_modelSD)
```

```
##
## Call:
## lm(formula = diff_obs ~ diff_pred, data = datPlotDE)
##
## Residuals:
##       Min        1Q    Median        3Q       Max
## -0.071991 -0.006301  0.003470  0.017260  0.065605
##
## Coefficients:
##             Estimate Std. Error t value Pr(>|t|)
## (Intercept)  0.02685    0.02422   1.108    0.304
## diff_pred    0.54087    0.33000   1.639    0.145
##
## Residual standard error: 0.04393 on 7 degrees of freedom
## Multiple R-squared:  0.2773, Adjusted R-squared:  0.1741
## F-statistic: 2.686 on 1 and 7 DF,  p-value: 0.1452
```

```r
confint(W_modelSD)
```

```
##                   2.5 %     97.5 %
## (Intercept) -0.03043303 0.08413255
## diff_pred   -0.23946801 1.32120619
```

```r
linearHypothesis(W_modelSD, c("(Intercept) = 0", "diff_pred = 1"), test = "Chisq")
```

```
## Linear hypothesis test
##
## Hypothesis:
## (Intercept) = 0
## diff_pred = 1
##
## Model 1: restricted model
## Model 2: diff_obs ~ diff_pred
##
##   Res.Df      RSS Df Sum of Sq  Chisq Pr(>Chisq)
## 1      9 0.017243
## 2      7 0.013508  2 0.0037353 1.9357     0.3799
```

```r
#### No outlier ####
outlier3D <- datPlotDE %>%
  filter(diff %in% boxplot(datPlotDE$diff, plot = FALSE)$out)

#### Figure D ####
plotSI2D <- datPlotDE %>%
  ggplot(aes(x = diff_pred, y = diff_obs, colour = Vaccine)) +
  geom_segment(aes(x=-0.05,xend=0.2,y=-0.05,yend=0.2),
               color="black",alpha=.7,lwd=0.5,lty=3) +
  geom_smooth(method='lm', color="gray80" ,formula=y~x,
              alpha=0.3, lwd=.6, fullrange=T,
              linetype="blank", show.legend=F) +
  annotate(geom = "text", x=0.175, y =0.19,
           label = "1:1 line", angle = 45, size = 2) +
  geom_point(size = 2, alpha = 0.8) + theme_classic() +
  scale_colour_manual(values = c("#004488","#DDAA33"),
            labels = c("Non-vaccine", "Mixed"),
            name = "Composition") +
  theme(legend.position = "none",
        axis.title = element_text(size = 8),
        axis.text = element_text(size = 6, colour = "black")) +
  xlab("Predicted Prevalence Change (NFDS)")+
  ylab("Observed Prevalence Change") +
  coord_fixed(ratio = 1, xlim=c(-0.05,0.2), ylim=c(-0.05,0.2)) +
  annotate("text",x=-0.05,y=0.19, size=1.5,hjust = 0,
           label=paste("SSE = ", sseE, "\nRMSE = ",
                       rmseE, "\nAdj. R2 = ", ars))

#### Figure E ####
datPlotDE <- datPlotDE %>%
  mutate(prorata = SC_freq_E1/sum(SC_freq_E1)) %>%
  mutate(diff_predPro =  prorata - SC_freq_E1)

stats <- summary(lm(datPlotDE$diff_predPro~datPlotDE$diff_obs))
ars <- round(stats$adj.r.squared, digits = 3)
sseE <- round(sse(datPlotDE$diff_obs,
                  datPlotDE$diff_predPro), digits = 3)
maeE <- round(mae(datPlotDE$diff_obs,
                  datPlotDE$diff_predPro),digits = 3)##Mean Absolute Error
rmseE <- round(rmse(datPlotDE$diff_obs,
                    datPlotDE$diff_predPro),digits = 3) ##Root Mean Squared Error

W_modelSE <- lm(data=datPlotDE,diff_obs~diff_predPro);
summary(W_modelSE)
```

```
##
## Call:
## lm(formula = diff_obs ~ diff_predPro, data = datPlotDE)
##
## Residuals:
##      Min       1Q   Median       3Q      Max
## -0.07442 -0.01786 -0.00284  0.04736  0.05935
##
## Coefficients:
```

```
##              Estimate Std. Error t value Pr(>|t|)
## (Intercept)   0.04741    0.02675   1.772     0.12
## diff_predPro  0.18930    0.35483   0.533     0.61
##
## Residual standard error: 0.05065 on 7 degrees of freedom
## Multiple R-squared:  0.03907,    Adjusted R-squared:  -0.0982
## F-statistic: 0.2846 on 1 and 7 DF,  p-value: 0.6102
```

```
confint(W_modelSE)
```

```
##                    2.5 %    97.5 %
## (Intercept)  -0.01584975 0.1106683
## diff_predPro -0.64974458 1.0283477
```

```
linearHypothesis(W_modelSE, c("(Intercept) = 0", "diff_predPro = 1"), test = "Chisq")
```

```
## Linear hypothesis test
##
## Hypothesis:
## (Intercept) = 0
## diff_predPro = 1
##
## Model 1: restricted model
## Model 2: diff_obs ~ diff_predPro
##
##   Res.Df      RSS Df Sum of Sq Chisq Pr(>Chisq)
## 1      9 0.031356
## 2      7 0.017961  2  0.013394  5.22    0.07353 .
## ---
## Signif. codes:  0 '***' 0.001 '**' 0.01 '*' 0.05 '.' 0.1 ' ' 1
```

```
plotSI2E <- datPlotDE %>% ggplot(aes(x = diff_predPro,
             y = diff_obs, colour = Vaccine)) +
  geom_segment(aes(x=-0.05,xend=0.2,y=-0.05,yend=0.2),
              color="black",alpha=.7,lwd=0.5,lty=3) +
  geom_smooth(method='lm', color="gray80" ,formula=y~x,
             alpha=0.3, lwd=.6, fullrange=T,
             linetype="blank", show.legend=F) +
  annotate(geom = "text", x=0.175, y =0.19,
          label = "1:1 line", angle = 45, size = 2) +
  geom_point(size = 2, alpha = 0.8) + theme_classic() +
  scale_colour_manual(values = c("#004488","#DDAA33"),
                     labels = c("Non-vaccine", "Mixed"),
                    name = "Composition") +
  theme(legend.position = "none",
       axis.title = element_text(size = 8),
       axis.text = element_text(size = 6, colour = "black")) +
  xlab("Predicted Prevalence Change (Pro rata)")+
  ylab("Observed Prevalence Change") +
  coord_fixed(ratio = 1, xlim=c(-0.05,0.2), ylim=c(-0.05,0.2)) +
  annotate("text",x=-0.05,y=0.19, size=1.5,hjust = 0,
          label=paste("SSE = ", sseE, "\nRMSE = ",
                      rmseE, "\nAdj. R2 = ", ars))

figureS2AB <- plot_grid(plotSI2A, plotSI2B, labels = c("A","B"),
                       ncol = 2, label_size = 10)
```

```
figureS2CE <- plot_grid(plotSI2C, plotSI2D, plotSI2E, label_size = 10,
                        nrow = 1, labels=c("C","D","E"))

figureS2 <- plot_grid(figureS2AB, figureS2CE, ncol=1)

setwd("PLOS_final_version_figures")
ggsave("SI_Fig2.png", figureS2, width = 19, height = 19/2, units = "cm")
ggsave("SI_Fig2.pdf", figureS2, width = 19, height = 19/2, units = "cm")
ggsave("SI_Fig2.tiff", figureS2, width = 19, height = 19/2, units = "cm")

listFigS2 <- list(figure_S2_A = datPlotA, figure_S2_B = datPlotB,
                  figure_S2_B_iter = dfpreMA_NVT_all_it,
                  figure_S2_C = datPlotC, figure_S2_DE = datPlotDE)
openxlsx::write.xlsx(listFigS2, file = "SI_Fig2_data.xlsx")
```

Figure S3: Core versus Accessory Genome distances

```
PA_Matrix_MD <- read_csv("data_southwestUS.csv")

#Accessory genome phylogeny
AG.tree <- read.tree("RAxML_bestTree.All.Binary.tre") #All

#Creating distance matrix from Tee
PatristicDistMatrix <- cophenetic(AG.tree) #Creates matrix of patristic distances
PatristicDist <- as.dist(PatristicDistMatrix,diag = TRUE, upper = TRUE)

#Setting up group/SC assignments for between SC patristic distance
seq.labels <- as.data.frame(rownames(PatristicDistMatrix)) #Obtaining ordered taxa
colnames(seq.labels) <- "taxa"
clades <- as.data.frame(cbind(PA_Matrix_MD$FinalName,PA_Matrix_MD$BAPS2)) #All data set
colnames(clades) <- c("taxa","clade")
seq.labels$id  <- 1:nrow(clades) #adding row number to maintain order for sorting after merge
labels.clades <- merge(seq.labels, clades, by="taxa") #merging
labels.clades <- labels.clades[order(labels.clades$id), ] #ordering

#Creating Final Matrix
md <- meandist(PatristicDist, labels.clades$clade) #calculating mean distance between clades
md.matrix <- as.dist(md,diag = FALSE, upper = TRUE)
md.matrix <- as.matrix(md.matrix)
diag(md.matrix) <- NA

#Saving Accessory geneome distances in long format
PatristicDist.long <- as.data.frame(as.table(md.matrix))
PatristicDist.long <- PatristicDist.long[! (PatristicDist.long$Var1 == PatristicDist.long$Var2),]
PatristicDist.long <- PatristicDist.long[!is.na(PatristicDist.long$Freq),]
AccGenomeDistances <- PatristicDist.long

#### Figure A ####
#Core genome distances
CG.tree <- read.tree("RAxML_bestTree.All.Core.tre") #Core genome tree
```

```r
PatristicDistMatrix <- cophenetic(CG.tree) #patristic distances
PatristicDist <- as.dist(PatristicDistMatrix,diag = TRUE, upper = TRUE)
#Setting up group/SC assignments for between SC patristic distance
seq.labels <- as.data.frame(rownames(PatristicDistMatrix)) #Obtaining ordered taxa
colnames(seq.labels) <- "taxa"
clades <- as.data.frame(cbind(df$FinalName,df$SC)) #All data set
colnames(clades) <- c("taxa","clade")
seq.labels$id  <- 1:nrow(clades) #adding row number to maintain order for sorting after merge
labels.clades <- merge(seq.labels, clades, by="taxa") #merging
labels.clades <- labels.clades[order(labels.clades$id), ] #ordering

md <- meandist(PatristicDist, labels.clades$clade) #calculating mean distance between clades
md.matrix <- as.dist(md,diag = FALSE, upper = TRUE)
md.matrix <- as.matrix(md.matrix)
diag(md.matrix) <- NA

#Accessory geneome distances
PatristicDist.long <- as.data.frame(as.table(md.matrix))
PatristicDist.long <- PatristicDist.long[! (PatristicDist.long$Var1 == PatristicDist.long$Var2),]
PatristicDist.long <- PatristicDist.long[!is.na(PatristicDist.long$Freq),]
CoreGenomeDistances <- PatristicDist.long

MergedDistances <- as.data.frame(cbind(CoreGenomeDistances,AccGenomeDistances))
colnames(MergedDistances) <- c("Var1a", "Var2b", "PCore", "Var1c", "Var2d","PAcc")

MergedDistances$MSE  <- (MergedDistances$PCore-MergedDistances$PAcc)^2
MergedDistancesMedian <- MergedDistances[(MergedDistances$PCore > 0.06 &
                                          MergedDistances$PCore < 0.15),]
MergedDistancesMedian <- within(MergedDistancesMedian,
                  A.quartile <- as.integer(cut(MergedDistancesMedian$PAcc,
                                quantile(MergedDistances$PAcc, probs=0:4/4), include.lowest=TRUE)))
MergedDistancesMedian <- within(MergedDistancesMedian,
                  P.quartile <- as.integer(cut(MergedDistancesMedian$PCore,
                                quantile(MergedDistances$PCore, probs=0:4/4), include.lowest=TRUE)))

model <- lm(MergedDistancesMedian$PCore~MergedDistancesMedian$PAcc)
car::outlierTest(model)
```

```
## No Studentized residuals with Bonferroni p < 0.05
## Largest |rstudent|:
##      rstudent unadjusted p-value Bonferroni p
## 207 -3.554855         0.00039412      0.43826
```

```r
MergedDistancesMedian$residuals <- abs(resid(model)) #Risiduals
MergedDistancesMedian$cooks <- cooks.distance(model)

#Make name varianble
MergedDistancesMedian$comp <- paste(MergedDistancesMedian$Var1a,
                                    MergedDistancesMedian$Var2b, sep = "-")

R2 <- round(cor(MergedDistancesMedian$PCore,
                MergedDistancesMedian$PAcc,method = "pearson"),2)

#### Plot A ####
```

```r
MergedDistancesMedian <- MergedDistancesMedian %>%
  left_join(rename(vaccineT, Var1a = SC, vaccine1a = Vaccine)) %>%
  left_join(rename(vaccineT, Var2b = SC, vaccine2b = Vaccine)) %>%
  unite("Composition", vaccine1a:vaccine2b, sep = " and ") %>%
  mutate(Composition = recode(Composition,
              "Vaccine type and Mixed type" =
                "Mixed type and Vaccine type")) %>%
  mutate(Composition = recode(Composition,
              "Non-vaccine type and Mixed type" =
                "Mixed type and Non-vaccine type")) %>%
  mutate(Composition = recode(Composition,
              "Non-vaccine type and Vaccine type" =
                "Vaccine type and Non-vaccine type"))

lev <- sort(unique(MergedDistancesMedian$Composition), decreasing = TRUE)

datPlotA <- MergedDistancesMedian %>%
  mutate(Composition = factor(Composition, levels = lev)) %>%
  select(PCore,PAcc,Composition) %>% distinct()

plotSI3A <-  datPlotA %>%
  ggplot(aes(PCore,PAcc)) + theme_classic() +
  geom_point(aes(col=Composition), size=1, alpha = 0.75) +
  scale_color_brewer(palette = "Dark2", drop=FALSE) +
  geom_density_2d(color="black", alpha=0.5, show.legend =FALSE) +
  geom_smooth(color="#636363", method=lm, alpha=.3, linetype="dashed",
              size=.6, formula = y~x,  show.legend=FALSE, se=TRUE) +
  labs(x="Core Genome Divergence (Patristic Distance)") + ylim(0.75,2.75) +
  labs(y="Accessory Genome Divergence \n(Patristic Distance)") +
  annotate("text", x = 0.12, y = 2.5, size = 2,
           label = paste("R^2 == ", R2), parse=TRUE) +
  theme(axis.title = element_text(size = 7),
        axis.text = element_text(colour = "black", size = 6),
        legend.position = c(0.05, 1),
        legend.justification = c(0.05, 1),
        legend.title = element_text(face="bold", size = 5),
        legend.text  = element_text(size = 4),
        legend.key.size = unit(0.3, "lines"),
        legend.background = element_rect(fill=NA, colour = NA))

#### Figure B ####
datPlot1A <- read_excel("PLOS_final_version_figures/Fig3_data.xlsx",1) %>%
  filter(SC!="01")

RF.E1.distmat <- as.matrix(dist(datPlot1A$r))
RF.E1.distmat[upper.tri(RF.E1.distmat)] <- NA; diag(RF.E1.distmat) <- NA
rownames(RF.E1.distmat) <- datPlot1A$SC
colnames(RF.E1.distmat) <- datPlot1A$SC
RF.E1.distlong <- na.omit(as.data.frame.table(RF.E1.distmat))
colnames(RF.E1.distlong) <- c("Var1a","Var2b","RelFit")

datPlotBC <- merge(RF.E1.distlong, MergedDistances, by=c("Var1a", "Var2b")) %>%
  select(Var1a, Var2b, PCore, PAcc, RelFit)
```

```r
#### Figure B ####

datPlotBC <- datPlotBC %>%
  left_join(rename(vaccineT, Var1a = SC, vaccine1a = Vaccine)) %>%
  left_join(rename(vaccineT, Var2b = SC, vaccine2b = Vaccine)) %>%
  unite("Composition", vaccine1a:vaccine2b, sep = " and ") %>%
  mutate(Composition = recode(Composition,
            "Vaccine type and Mixed type" =
              "Mixed type and Vaccine type")) %>%
  mutate(Composition = recode(Composition,
            "Non-vaccine type and Mixed type" =
              "Mixed type and Non-vaccine type")) %>%
  mutate(Composition = recode(Composition,
            "Non-vaccine type and Vaccine type" =
              "Vaccine type and Non-vaccine type")) %>%
  mutate(Composition = factor(Composition, levels = lev))

plotSI3B <- datPlotBC %>% select(Composition, PCore, RelFit) %>%
  distinct() %>% ggplot(aes(PCore,RelFit)) +
  geom_point(aes(col=Composition), size=1, alpha = 0.75) +
  scale_color_brewer(palette = "Dark2", drop=FALSE) +
  geom_density_2d(color="black", alpha=0.5, show.legend =FALSE) +
  labs(x="Core Genome Divergence (Patristic Distance)") +
  labs(y="Absolute fitness difference") + theme_classic() +
  theme(axis.title = element_text(size = 7),
        axis.text = element_text(colour = "black", size = 6),
        legend.position = c(0.05, 1),
        legend.justification = c(0.05, 1),
        legend.title = element_text(face="bold", size = 5),
        legend.text  = element_text(size = 4),
        legend.key.size = unit(0.3, "lines"),
        legend.background = element_rect(fill=NA, colour = NA))


plotSI3C <- datPlotBC %>% select(Composition, PAcc, RelFit) %>%
  distinct() %>% ggplot(aes(PAcc, RelFit)) +
  geom_point(aes(col=Composition), size=1, alpha = 0.75) +
  scale_color_brewer(palette = "Dark2", drop = FALSE) +
  geom_density_2d(color="black", alpha=0.5, show.legend =FALSE) +
  labs(x="Accessory Genome Divergence (Patristic Distance)") +
  labs(y="Absolute fitness difference") + theme_classic() +
  theme(axis.title = element_text(size = 7),
        axis.text = element_text(colour = "black", size = 6),
        legend.position = c(0.05, 1),
        legend.justification = c(0.05, 1),
        legend.title = element_text(face="bold", size = 5),
        legend.text  = element_text(size = 4),
        legend.key.size = unit(0.3, "lines"),
        legend.background = element_rect(fill=NA, colour = NA))

figureS3 <- plot_grid(ncol=3, plotSI3A, plotSI3B, plotSI3C,
                      labels = "AUTO", align = 'h', label_size = 10)
```

```r
setwd("PLOS_final_version_figures")
ggsave("SI_Fig3.png", figureS3, width = 19, height = 6, units = "cm")
ggsave("SI_Fig3.pdf", figureS3, width = 19, height = 6, units = "cm")
ggsave("SI_Fig3.tiff", figureS3, width = 19, height = 6, units = "cm")

listFigS3 <- list(figure_S3_A = datPlotA, figure_S3_BC = datPlotBC)
openxlsx::write.xlsx(listFigS3, file = "SI_Fig3_data.xlsx")
```

Figure S4: Distribution of COGs by SCs

```r
SC_freq_E1 <- SC_freq_postV_obs %>% select(SC, PCV7.actual) %>%
  left_join(df) %>% subset(Epoch1 == "E1") %>%
  select(SC, HMPREF0837_12128:HMPREF0837_10616) %>%
  arrange(SC) %>% group_by(SC) %>%
  summarise_at(vars(HMPREF0837_12128:HMPREF0837_10616),mean) %>%
  ungroup() %>%
  pivot_longer(cols=HMPREF0837_12128:HMPREF0837_10616,
               names_to = "COG", values_to = "f") %>%
  mutate(Epoch = "Pre-vaccine")

SC_freq_E3 <- SC_freq_postV_obs %>% select(SC, PCV7.actual) %>%
  left_join(df) %>% subset(Epoch1 == "E3") %>%
  select(SC, HMPREF0837_12128:HMPREF0837_10616) %>%
  arrange(SC) %>% group_by(SC) %>%
  summarise_at(vars(HMPREF0837_12128:HMPREF0837_10616),mean) %>%
  ungroup() %>%
  pivot_longer(cols=HMPREF0837_12128:HMPREF0837_10616,
               names_to = "COG", values_to = "f") %>%
  mutate(Epoch = "Post-vaccine")

datPlot <- bind_rows(SC_freq_E1, SC_freq_E3)

plotSI4 <- datPlot %>% ggplot(aes(f, fill = Epoch)) +
  geom_histogram(aes(y = ..count..), position = "dodge", bins = 10) +
  facet_wrap(~SC, nrow = 3) +
  xlab("\n Accessory gene frequency") + theme_minimal() +
  scale_fill_manual("", values = c("#D3723D", "#8A9DA4")) +
  annotate("segment", x=-Inf, xend=Inf, y=-Inf, yend=-Inf)+
  annotate("segment", x=-Inf, xend=-Inf, y=-Inf, yend=Inf) +
  scale_x_continuous(breaks=seq(0,1,0.2)) +
  theme(axis.title = element_text(size = 7),
        strip.text = element_text(size = 6),
        legend.position = "bottom",
        legend.key.size = unit(0.6, "lines"),
        legend.title = element_blank(),
        legend.text = element_text(size = 6),
        axis.text = element_text(size = 5, colour = "black"))

setwd("PLOS_final_version_figures")
ggsave("SI_Fig4.png", plotSI4, width = 19, height = 8, units = "cm")
ggsave("SI_Fig4.pdf", plotSI4, width = 19, height = 8, units = "cm")
```

```
ggsave("SI_Fig4.tiff", plotSI4, width = 19, height = 8, units = "cm")
openxlsx::write.xlsx(datPlot, file = "SI_Fig4_data.xlsx")
```

Table 1: Statistics

```
#### Accessory genome SA ####
## Sensitivity analysis using a subsample of 119 isolates
## collected in 2010 prior to the initiation of PCV13

#### Data ####
dfSenst <- df %>% separate(FinalName, c("ID1", "ID2", "ID3", "Year"))
dfSenstE12 <- dfSenst %>% filter(Epoch1 != "E3")
dfSenstE3 <- dfSenst %>% filter(Epoch1 == "E3" & Year == "2010")
dfS <- bind_rows(dfSenstE12,dfSenstE3)

dfFVTS <- dfS %>% select(SC,PCV7.actual, Epoch1) %>%
  group_by(Epoch1) %>%  count(SC,PCV7.actual) %>%
  mutate(freq = round(prop.table(n), digits = 3)) %>% ungroup() %>%
  select(Epoch1, SC, PCV7.actual, freq) %>%
  spread(Epoch1, freq, fill = 0) %>% arrange(SC,PCV7.actual)

#### Present at E1 ####
SCE1S <- dfFVTS %>% subset(E1 > 0) %>% ## & SC !="27"
  select(SC, PCV7.actual) %>%
  mutate(Epoch1 = "E1")

#### NVTs Present at E1 ####
SCE2S <- SCE1S %>% subset(PCV7.actual == "NVT"  & SC !="27")

SC_freq_dfS <- dfS %>% select(SC, PCV7.actual, Epoch1,
                              HMPREF0837_12128:HMPREF0837_10616) %>%
  arrange(SC) %>% group_by(SC,PCV7.actual,Epoch1) %>%
  mutate(SC_n = n()) %>% ungroup() %>%
  group_by(SC,PCV7.actual,Epoch1,SC_n) %>%
  summarise_at(vars(HMPREF0837_12128:HMPREF0837_10616),mean) %>%
  ungroup()

### Get the matrix and the SC for the pre-vaccine epch "E1"
df_preVS <- SCE1S %>% left_join(SC_freq_dfS)
SC_freq_preVS <- as.matrix(df_preVS %>%
                 mutate(SC_freq=SC_n/sum(SC_n)) %>% select(SC_freq))
SC_COG_preVS <- as.matrix(t(df_preVS %>%
                 select(HMPREF0837_12128:HMPREF0837_10616)))

#### Get e_l ####
el_S <- SC_COG_preVS %*% SC_freq_preVS

df_postVS <- SCE2S %>% left_join(SC_freq_dfS)
SC_COG_postVS <- as.matrix(t(df_postVS %>%
                 select(HMPREF0837_12128:HMPREF0837_10616)))
```

```r
SC_freq_postV_obsS <- SCE2S %>% mutate(Epoch1 = "E3") %>%
  left_join(SC_freq_dfS) %>%
  mutate(SC_freq=SC_n/sum(SC_n, na.rm = T)) %>%
  select(SC, PCV7.actual, SC_freq) %>%
  mutate(SC_freq = replace_na(SC_freq, 0))

## Predict postV frequencies
SC_freq_postV_predS <- QP(SC_COG_postVS, el_S) #Matrix: rows = COGs, columns = (SCs - VT)
SC_freq_postV_obsS <- SC_freq_postV_obsS %>%
  mutate(SC_pred = SC_freq_postV_predS)

SC_freq_E1S <- df_preVS %>%
  mutate(SC_freq_E1=SC_n/sum(SC_n)) %>%
  select(SC,PCV7.actual,SC_freq_E1)

SC_freq_postV_diffS <- SC_freq_postV_obsS %>%
  left_join(SC_freq_E1S) %>%
  mutate(diff_pred = SC_freq_E1 - SC_pred,
         diff_obs = SC_freq_E1 - SC_freq)

SC_freq_postV_diffS <- SC_freq_postV_diffS %>%
  mutate(diff = abs(diff_pred - diff_obs)) %>%
  arrange(diff)

stats <- summary(lm(SC_freq_postV_diffS$diff_pred~SC_freq_postV_diffS$diff_obs))
ars <- round(stats$adj.r.squared, digits = 3)
sseE <- round(sse(SC_freq_postV_diffS$diff_obs,
                  SC_freq_postV_diffS$diff_pred), digits = 3)
rmseE <- round(rmse(SC_freq_postV_diffS$diff_obs,
                    SC_freq_postV_diffS$diff_pred),digits = 3)

accNFDSSA <- data.frame(Model = "Accesory genome (NFDS) SA",
                        nloci = length(el_S), adj.r.squared = ars,
                        SSE = sseE, RMSE = rmseE)

SC_freq_postV_diffS <- SC_freq_postV_diffS %>%
  mutate(prorata = SC_freq_E1/sum(SC_freq_E1)) %>%
  mutate(diff_predPro = SC_freq_E1 - prorata) %>%
  mutate(diffP = abs(diff_predPro - diff_obs))

stats <- summary(lm(SC_freq_postV_diffS$diff_predPro~SC_freq_postV_diffS$diff_obs))
ars <- round(stats$adj.r.squared, digits = 3)
sseE <- round(sse(SC_freq_postV_diffS$diff_obs,
                  SC_freq_postV_diffS$diff_predPro), digits = 3)
rmseE <- round(rmse(SC_freq_postV_diffS$diff_obs,
                    SC_freq_postV_diffS$diff_predPro),digits = 3)

accProrataSA <- data.frame(Model = "Accesory genome (Prorata) SA",
                           nloci = length(el_S), adj.r.squared = ars,
                           SSE = sseE, RMSE = rmseE)

#####################
```

```r
#### Core genome ####
dfcore <- read.csv("CoreSNPpresenceAbsence.txt", header = F)
dfcore <- dfcore %>% as_tibble() %>% rename(FinalName = V1)
dfcore <- df %>% select(FinalName, SC, PCV7.actual, Epoch1) %>%
  left_join(dfcore)

dfFVTcore <- dfcore %>% select(SC,PCV7.actual, Epoch1) %>%
  group_by(Epoch1) %>%  count(SC,PCV7.actual) %>%
  mutate(freq = round(prop.table(n), digits = 3)) %>% ungroup() %>%
  select(Epoch1, SC, PCV7.actual, freq) %>%
  spread(Epoch1, freq, fill = 0) %>% arrange(SC,PCV7.actual)

#### Present at E1 ####
SCE1core <- dfFVTcore %>% subset(E1 > 0) %>% ## & SC !="27"
  select(SC, PCV7.actual) %>%
  mutate(Epoch1 = "E1")

#### NVTs Present at E1 ####
SCE2core <- SCE1core %>% subset(PCV7.actual == "NVT"  & SC !="27")


SC_freq_dfcore <- dfcore %>% select(SC, PCV7.actual, Epoch1,
                                   V2:V62654) %>%
  arrange(SC) %>% group_by(SC,PCV7.actual,Epoch1) %>%
  mutate(SC_n = n()) %>% ungroup() %>%
  group_by(SC,PCV7.actual,Epoch1,SC_n) %>%
  summarise_at(vars(V2:V62654),mean) %>%
  ungroup()

### Get the matrix and the SC for the pre-vaccine epch "E1"
df_preVcore <- SCE1core %>% left_join(SC_freq_dfcore)
SC_freq_preVcore <- as.matrix(df_preVcore %>%
                    mutate(SC_freq=SC_n/sum(SC_n)) %>% select(SC_freq))
SC_COG_preVcore <- as.matrix(t(df_preVcore %>% select(V2:V62654)))

#### Get e_l for the core (62653 loci) ####
el_core <- SC_COG_preVcore %*% SC_freq_preVcore

#### Observed versus prediced prevalence ####
df_postVcore <- SCE2core %>% left_join(SC_freq_dfcore)
SC_COG_postVcore <- as.matrix(t(df_postVcore %>% select(V2:V62654)))

SC_freq_postV_obsCore <- SCE2core %>% mutate(Epoch1 = "E3") %>%
  left_join(SC_freq_dfcore) %>%
  mutate(SC_freq=SC_n/sum(SC_n, na.rm = T)) %>%
  select(SC, PCV7.actual, SC_freq) %>%
  mutate(SC_freq = replace_na(SC_freq, 0))

## Predict postV frequencies
SC_freq_postV_predCore <- QP(SC_COG_postVcore, el_core)
SC_freq_postV_obsCore <- SC_freq_postV_obsCore %>%
  mutate(SC_pred = SC_freq_postV_predCore)
```

```
SC_freq_E1core <- df_preVcore %>%
  mutate(SC_freq_E1=SC_n/sum(SC_n)) %>%
  select(SC,PCV7.actual,SC_freq_E1)

SC_freq_postV_diffcore <- SC_freq_postV_obsCore %>%
  left_join(SC_freq_E1core) %>%
  mutate(diff_pred = SC_freq_E1 - SC_pred,
         diff_obs = SC_freq_E1 - SC_freq)

SC_freq_postV_diffcore <- SC_freq_postV_diffcore %>%
  mutate(diff = abs(diff_pred - diff_obs)) %>%
  arrange(diff)

#### Pro rata ####
SC_freq_postV_diffcore <- SC_freq_postV_diffcore %>%
  mutate(prorata = SC_freq_E1/sum(SC_freq_E1)) %>%
  mutate(diff_predPro = SC_freq_E1 - prorata) %>%
  mutate(diffP = abs(diff_predPro - diff_obs))

#### Stats core NFDS ####
stats <- summary(lm(SC_freq_postV_diffcore$diff_pred~SC_freq_postV_diffcore$diff_obs))
ars <- round(stats$adj.r.squared, digits = 3)
sseE <- round(sse(SC_freq_postV_diffcore$diff_obs,
                  SC_freq_postV_diffcore$diff_pred), digits = 3)
rmseE <- round(rmse(SC_freq_postV_diffcore$diff_obs,
                    SC_freq_postV_diffcore$diff_pred),digits = 3)

coreNFDS <- data.frame(Model = "Core genome (NFDS)", nloci = length(el_core),
                       adj.r.squared = ars, SSE = sseE, RMSE = rmseE)

#### Stats core Pro rata ####
stats <- summary(lm(SC_freq_postV_diffcore$diff_predPro~SC_freq_postV_diffcore$diff_obs))
ars <- round(stats$adj.r.squared, digits = 3)
sseE <- round(sse(SC_freq_postV_diffcore$diff_obs,
                  SC_freq_postV_diffcore$diff_predPro), digits = 3)
rmseE <- round(rmse(SC_freq_postV_diffcore$diff_obs,
                    SC_freq_postV_diffcore$diff_predPro),digits = 3)

coreProrata <- data.frame(Model = "Core genome (Pro rata)", nloci = length(el_core),
                          adj.r.squared = ars, SSE = sseE, RMSE = rmseE)

##############################

#### Metabolic loci ####
dfmeta <- read.csv("Core_Metabolic_SNPpresenceAbsence.txt", header = F)
dfmeta <- dfmeta %>% as_tibble() %>% rename(FinalName = V1)
dfmeta <- df %>% select(FinalName, SC, PCV7.actual, Epoch1) %>%
  left_join(dfmeta)

dfFVTmeta <- dfmeta %>% select(SC,PCV7.actual, Epoch1) %>%
  group_by(Epoch1) %>%  count(SC,PCV7.actual) %>%
  mutate(freq = round(prop.table(n), digits = 3)) %>% ungroup() %>%
  select(Epoch1, SC, PCV7.actual, freq) %>%
```

```r
  spread(Epoch1, freq, fill = 0) %>% arrange(SC,PCV7.actual)

#### Present at E1 ####
SCE1meta <- dfFVTmeta %>% subset(E1 > 0) %>% ## & SC !="27"
  select(SC, PCV7.actual) %>%
  mutate(Epoch1 = "E1")

#### NVTs Present at E1 ####
SCE2meta <- SCE1meta %>% subset(PCV7.actual == "NVT"  & SC !="27")


SC_freq_dfmeta <- dfmeta %>% select(SC, PCV7.actual, Epoch1,
                                    V2:V22434) %>%
  arrange(SC) %>% group_by(SC,PCV7.actual,Epoch1) %>%
  mutate(SC_n = n()) %>% ungroup() %>%
  group_by(SC,PCV7.actual,Epoch1,SC_n) %>%
  summarise_at(vars(V2:V22434),mean) %>%
  ungroup()

### Get the matrix and the SC for the pre-vaccine epch "E1"
df_preVmeta <- SCE1meta %>% left_join(SC_freq_dfmeta)
SC_freq_preVmeta <- as.matrix(df_preVmeta %>%
                    mutate(SC_freq=SC_n/sum(SC_n)) %>% select(SC_freq))
SC_COG_preVmeta <- as.matrix(t(df_preVmeta %>% select(V2:V22434)))

#### Get e_l for the meta (62653 loci) ####
el_meta <- SC_COG_preVmeta %*% SC_freq_preVmeta

#### Observed versus prediced prevalence ####
df_postVmeta <- SCE2meta %>% left_join(SC_freq_dfmeta)
SC_COG_postVmeta <- as.matrix(t(df_postVmeta %>% select(V2:V22434)))

SC_freq_postV_obsMeta <- SCE2meta %>% mutate(Epoch1 = "E3") %>%
  left_join(SC_freq_dfmeta) %>%
  mutate(SC_freq=SC_n/sum(SC_n, na.rm = T)) %>%
  select(SC, PCV7.actual, SC_freq) %>%
  mutate(SC_freq = replace_na(SC_freq, 0))

## Predict postV frequencies
SC_freq_postV_predMeta <- QP(SC_COG_postVmeta, el_meta)
SC_freq_postV_obsMeta <- SC_freq_postV_obsMeta %>%
  mutate(SC_pred = SC_freq_postV_predMeta)

SC_freq_E1meta <- df_preVmeta %>%
  mutate(SC_freq_E1=SC_n/sum(SC_n)) %>%
  select(SC,PCV7.actual,SC_freq_E1)

SC_freq_postV_diffmeta <- SC_freq_postV_obsMeta %>%
  left_join(SC_freq_E1meta) %>%
  mutate(diff_pred = SC_freq_E1 - SC_pred,
         diff_obs = SC_freq_E1 - SC_freq)

SC_freq_postV_diffmeta <- SC_freq_postV_diffmeta %>%
```

```r
  mutate(diff = abs(diff_pred - diff_obs)) %>%
  arrange(diff)

#### Pro rata ####
SC_freq_postV_diffmeta <- SC_freq_postV_diffmeta %>%
  mutate(prorata = SC_freq_E1/sum(SC_freq_E1)) %>%
  mutate(diff_predPro = SC_freq_E1 - prorata) %>%
  mutate(diffP = abs(diff_predPro - diff_obs))

#### Stats metabolic loci NFDS ####
stats <- summary(lm(SC_freq_postV_diffmeta$diff_pred~SC_freq_postV_diffmeta$diff_obs))
ars <- round(stats$adj.r.squared, digits = 3)
sseE <- round(sse(SC_freq_postV_diffmeta$diff_obs,
                  SC_freq_postV_diffmeta$diff_pred), digits = 3)
rmseE <- round(rmse(SC_freq_postV_diffmeta$diff_obs,
                    SC_freq_postV_diffmeta$diff_pred),digits = 3)

metaNFDS <- data.frame(Model = "Metabolic loci (NFDS)", nloci = length(el_meta),
                       adj.r.squared = ars, SSE = sseE, RMSE = rmseE)

#### Stats metabolic loci Pro rata ####
stats <- summary(lm(SC_freq_postV_diffmeta$diff_predPro~SC_freq_postV_diffmeta$diff_obs))
ars <- round(stats$adj.r.squared, digits = 3)
sseE <- round(sse(SC_freq_postV_diffmeta$diff_obs,
                  SC_freq_postV_diffmeta$diff_predPro), digits = 3)
rmseE <- round(rmse(SC_freq_postV_diffmeta$diff_obs,
                    SC_freq_postV_diffmeta$diff_predPro),digits = 3)

metaProrata <- data.frame(Model = "Metabolic loci (Pro rata)", nloci = length(el_meta),
                          adj.r.squared = ars, SSE = sseE, RMSE = rmseE)

#############################

#### create table ####
table1 <- bind_rows(accNFDS, accProrata, accNFDSSA,
                   accProrataSA, coreNFDS, coreProrata,
                   metaNFDS, metaProrata)
setwd("PLOS_final_version_figures")
openxlsx::write.xlsx(table1, file = "table1.xlsx")
```
