# Supplementary material for: Frequency-dependent selection can forecast evolution in Streptococcus pneumoniae
Source: PLoS Biol. 2020 Oct 22;18(10):e3000878. doi: 10.1371/journal.pbio.3000878 (PMC7580979; doi:10.1371/journal.pbio.3000878)
Supplement: Attachment — Submitted filename: PBIOLOGY-S-20-00669_reviewer_response_Final.docx [file pbio.3000878.s012.docx]

**Reviewer #1: [identifies himself as Chris Illingworth]**

*Major comments:*

While the results shown are promising, my concern with this work is the extent to which the data are representative of S. pneumoniae populations in general. The evolution of a population comprises both changes arising from standing variation (e.g. changes in the frequencies of existing variants or strains in the population), and changes arising from de novo events (including the onset of new mutations, migration into the local environment, and so forth). In common with most approaches to evolutionary prediction, the model presented considers only the first kind of evolutionary change.

In the dataset shown, the initial population comprised 33 strains (Fig. 1), and 12 years later the same strains predominated with the loss only of two strains that were vaccinated against and the gain of only two more strains. The S. pneumoniae population considered is therefore something of a closed system. This leads to questions about the data used for the study.

1) To what extent are these data representative of S. pneumoniae populations in general? It is noted that the data were collected from Native American populations. Could these represent unusually closed populations in terms of the potential introduction of new strains from outside of the community? Would the same be true of bacterial populations in a major city? In general, how stable is a population against invasion on a timescale of 12 years?

**We thank the reviewer for their insightful comments. This was an important question we attempted to address with our previous publication of these data (Azarian, PLoS Pathog. 2018, PMID: 29617440). We conducted population genomic analysis of each strain and investigated the origin of two strains that were observed for the first time post-PCV7 in the Southwest US sample. We conjectured that one strain was likely not sampled but present pre-PCV7 while the other was likely introduced. Both of these strains were found during the same period in the Massachusetts sample, which we use here to estimate the “global fitness”. While the Southwest US sample might be epidemiologically considered a comparatively closed system, the dominant strains found in those communities are found throughout the US, although in different proportions depending on the location. This is true to a lesser extent of global samples (see Croucher et al 2017) especially when LMIC are considered. In general, this finding is observed when comparing populations - While the exact frequencies of strains in each location may vary, the dominant strains are widespread. We explain this is more detail in our response to Reviewer 2’s comments. In the revised version of the manuscript, we have clarified this point in the text and added citations for studies that investigated the global distribution of strains. Of course, the distribution of strains is specific to each bacteria (e.g. *Staphylococcus aureus, E. coli*); however, research in each respective field can contextualize the distribution of strains when attempting to apply the same method we introduce here.**

**Regarding the invasion potential and timescale for these events, it is an area of ongoing research that extends beyond the scope of the current analysis. In general, the emergence of new strains is difficult to assess because of the lack of consistent genomic surveillance in most locales. In the Southwest US samples, there is at least one strain that we believe emerged post-vaccine. There are indeed other examples of strains that emerged after the introduction of vaccine; however, even with coalescent and population genomic analysis, it is difficult to assess the timescale of these events. As more genomic data are generated based on ongoing surveillance studies, we will hopefully be able to assess the frequency and success of emerging strains. We hope that the method we introduce here can be used to assess the likelihood of invasion or emergence based on a strain’s predicted fitness.**

2) Why were these specific datasets chosen? It is mentioned that the second dataset is limited in the span between samples. Very many S. pneumoniae genomes have been sequenced; was the choice based simply on a lack of availability of data collected before and after a specific vaccination program?

**Even though there are considerable published genomic data available for *S. pneumoniae*, the present dataset is unique in four respects: 1) It includes the earliest cross-sectional sample of *S. pneumoniae* in a defined population, and it is the only sample that predates the introduction of PCV7; 2) Our study period, 15 years, is the longest duration of observation of any genomic sample; 3) As a site for PCV7 and PCV13 vaccine trials, the population is well characterized and the epidemiology of pneumococcal disease well understood; and 4) It is focused on carriage isolates and not invasive pneumococcal disease - Isolates from invasive disease only capture a biased sub-sample of the pneumococcal population because some strains are more invasive than others, and the relevant sample is therefore from carriage.** **We have detailed this decision in the methods section of the revised version of the manuscript.**

**Other existing genomic samples of *S. pneumoniae*** **suffer from** **critical limitations. One is not appropriate because there was no vaccine use (Maela). One does not include sufficient follow up to allow the population to recover (Southampton), or contained only invasive isolates (Netherlands), which would systematically inflate the frequencies of strains associated with disease and underestimate the frequencies of those that are not (see Brueggemann et al 2003) – making it impossible to meaningfully quantify the predictive capabilities of the method. They are also longitudinal samples rather than subsequent cross-sectional ones, which would be preferred to characterize the population at each time point. Therefore, we expect to diverge substantially from our predictive model. We hope that the publication of this approach will provide method for the community to evaluate high quality samples as they are collected.**

3) It is noted that a subset of the data from three studies was taken. Please note how this subset was chosen. My view is that the method presented here is of interest even if it is limited in application to unusually closed systems; what I am unsure of is the generality of its predictive value. Further comment should be made on the data.

**The samples were randomly selected from the three cross-sectional studies of pneumococcal carriage conducted among the Native American Communities, which assessed the impact of vaccination on carriage of vaccine serotypes. In our previous study of the pneumococcal phylodynamics in the context of vaccination, we assessed the distribution of serotypes in our sub-sample to the larger sample for which serotype data was available (Azarian, PLoS Pathog. 2018, PMID: 29617440). Indeed, our subsample captured serotype diversity in the parent study. Further, the sub-sample size was selected to capture low prevalence strains in the population. Further, in our current analysis we have assessed the population structure of the Native American sample in the context of the Massachusetts dataset described in the manuscript. When the population structure of both samples are compared, the distribution of strains is comparable, there are only two strains present in Mass that are not in the Native American sample (1.3% and 2.2% overall prevalence), two low prevalence (0.3%) strains present in the Native American sample that are not present in the Mass sample.**

*Minor comments:*

- There is an ambiguity regarding the composition of the individual strains. Line 153 mentions the frequency of a gene within a single strain. Is it the case that this frequency is predominantly either zero or one? If not, the model potentially implies that within-strain evolution would be expected in addition to the overall changes in strain frequency. Was this looked at?

**In the model, presence and absence of accessory genes were assessed as 0/1. With the empirical data, the presence of a gene is represented as the frequency of the COG among members of that strain. Further, due to recombination and the movement of mobile genetic elements, we do expect some level of intra-strain evolution, and we do observe some diversity of COG presence/absence within a strain. S5 Figure in the current version of the manuscript shows the stability of the COGs from pre- to post-vaccine. This also shows the distribution of COG frequencies pre-vaccine and then again at the post-vaccine equilibrium time-point that we predict.**

- In equation 1 I think that x_i is the frequency of strain i after the population has been rescaled to account for the removal of the vaccinated-against strains (i.e. the frequencies from the pro-rata model). This should be clarified.

**This is now clarified it in the main text (lines 161-163).**

- Would a combination of information from the combined and accessory genomes provide a better result than taking each set individually?

**For the joint analysis of Massachusetts and Southwest US datasets, a pangenome analysis of all strains was conducted. This is what was used for assessing the population structure. In general, sub-setting the datasets does not change the assignment of the accessory COG, it changes the core and accessory genome size.**

- In Figure S3 the fittest genotypes according to the accessory genome measure are significantly more diverged than the bulk of the population; is there a reason for this? To what extent does the divergence measure shown differ from the mean divergence from a vaccinated strain?

**We thank the review for this comment. S3 Figure shows the core/accessory genome divergence against the difference in fitness. Fitness was calculated by comparing the accessory genome of a strain to vaccine type strains as described in the methods. Figures S3B and S3C in particular illustrate that strains divergent in core and accessory genome distance, respectively, are also divergent in their fitness values. We assume that strains more closely related in terms of accessory genome would have similar fitness values. In general, this is the relationship we observe; however, there are outliers, which suggests that content is important, just not presence/absence. To make this clearer, we have revised the figure so that the points on the plot a colored by the strain vaccine type of the pairwise comparison (e.g., VT-VT, NVT-VT, NVT-Mixed, etc.).**

- Figure 1 is nice in showing the changes in different strains relative to the pro rata model; could a similar figure be created for the fitness-based model?

**We have included the below figure to address the reviewer’s immediate question, where open colored diamonds show the predicted changes, filled colored dots represent observed changes, and black dots and whiskers show the *pro rata* predicted values. There are several options for how data currently shown in Figure 3C and 3D can be presented, and this figure provides an alternative. However, the reason we decided on the current presentation is because the strain frequencies are different from what is shown in Figure 1, and we feel this would cause confusion, as readers would want to directly compare these frequencies. In the current version of the manuscript, we have made every attempt to clearly state which strains were included in each analysis. In addition to a detailed explanation in each figure legend, we have also included a supplemental table that states for each figure the data source, strains included, and explanation of sample.**

- The colors in Figure 1 were too similar for my liking. The contrast between blue and red is clear, but the purple is hard to distinguish from the blue, particularly given the two-tone nature of the chart. Please make this more distinct.

**We thank the referee for this observation. We have changed the purple for golden, using a palette that is also color-blind compatible.**

- "Enables to predict" in the abstract - please correct the grammar here.

**Thank you for identifying this omission. The sentence has been corrected to ‘Enables us to predict’.**

**Reviewer #2: [identifies himself as Kevin Bakker]**

Overall, this is an interesting study, however the authors need to be a bit more clear in which samples were used for each analysis and explain the impact of the work.

1) Figure 1 explains there are 35 strains in the study out of ~90 circulating, figure 2 randomly selects 35/90 strains, Figure 3A uses only 31 and it's not clear which of the 31 are used, and in Figs 3B-3D there are only 27 strains - it's not clear which 27 these are.

**We note that there appears to be some confusion between use of the terms ‘strains’ and ‘serotypes’ in our study. There are more than 90 serotypes of *Streptococcus pneumoniae* described, which are defined by the antigenic properties of their polysaccharide capsule. Sequence clusters, on the other hand, are statistically defined based on nucleotide or sequence variation in the genome and we refer to them here as ‘strains’ to reduce field-specific jargon. A strain (i.e., lineage/sequence cluster) may be comprised of multiple serotypes, which we have now clarified in the revised version of the manuscript (lines 95-6).**

**Regarding the analysis in Figure 1, the population structure of the Southwest US dataset was defined based on the entire sample and was intentionally maintained following our previous work (Azarian, PLoS Pathog. 2018, PMID: 29617440) so that publication could be referenced for more detail on each strain. That analysis defined 35 strains: some containing vaccine serotypes only, non-vaccine serotypes only, and other a mix of vaccine and non-vaccine serotypes. To be included in the analysis in Figure 3A, the strain had to have a non-vaccine component and be present before in introduction of the PCV7 vaccine (pre-vaccine). This was true for 31 strains. We have slightly updated the figure legend of Figure 3 to clarify this accordingly “*Predicted fitness was calculated using data solely from the pre-vaccine sample (n=31), with the exceptions of strains for which there were no non-vaccine serotype (NVT) isolates present in the sample before the introduction of PCV7 (n=4).*”**

**We thank the reviewer for pointing out the lack of clarity in the terminology used to describe vaccine periods in the manuscript. This confusion originated because there is a difference in vaccine introduction timing between the two samples. In addition, since no explicitly “pre-vaccine” sample exists from Massachusetts, we used an early post-PCV7 sample to represent the pre-vaccine time point. In the revised version, we have clarified and standardized the use of these terms throughout the manuscript. We added a supplemental figure (S4 Figure) that illustrates the terminology and the time periods for both Southwest US dataset and the Massachusetts dataset. In addition, we now include a supplemental table (S2 Table) that details which strains were used for each analysis/figure as well as an explanation of why some strains were omitted.**

2) There are multiple time scales discussed (6 years not long enough for evolution but 12 years is ok, 2001-2004 was a peri-vaccine for MA but 2006-2008 in the SW, whereas 2007 was post-vaccine in MA but 2010-2012 in the SW). Meanwhile in the results and discussion the SW dataset is partitioned into pre- and post-vaccine. My point being there are too many time period definitions throughout and it's extremely difficult to follow.

**We agree that it is sometimes difficult to track the time periods, the strains used in each analysis, and most importantly why they varied. Our primary focus is on the Southwest US dataset because it spans the longest duration of time, has a true pre-vaccine sample, and has temporally appropriate sampling to capture the impact of the vaccine. The Massachusetts dataset has several limitations, as noted in the response to reviewer #1; however, we felt it important to include a secondary validation dataset to demonstrate the robustness of the methods and results. A prominent limitation, which adds to the confusion reviewer 2 notes, is that its period of collection of begins after the introduction of PCV7 and ends before the population equilibrates. This was determined through extensive analysis of both datasets as detailed in our original manuscript on pneumococcal population genomics in the SW US (Azarian, PLoS Pathog. 2018, PMID: 29617440) and Massachusetts (Croucher, Nat Genetics, 2013, PMID: 23644493). We assigned the terms pre-vaccine or pre-PCV7 to describe the period before the introduction of vaccine. This period did not exist in the Massachusetts data because collection straddled the introduction of PCV7, hence “peri-vaccine” (Details in S2 Table and S4 Figure of the current version). We have made every effort to describe in detail which data were used for each analysis (lines 398-403).**

3) While interesting, the authors fail to note why this work is impactful or significant.

**Regarding the significance of this work, predicting how pathogens will evolve is a primary goal of population genomic studies – albeit one that has been mostly pursued in viral pathogens. For bacteria, an intervention targeting some but not all of the population may inadvertently lead to the emergence of virulent or antibiotic resistant strains. These data can then be used for vaccine development (e.g. to target new antigenic types or to strategically target strains that would lead to a more “perturbed” population) or to surveille emerging lineages (what we call strains here). In the revised version of the manuscript, we have articulated these points in more detail in the introduction and discussion.**

4) Are certain strains more pathogenic?

**Indeed, in terms of rates of invasive pneumococcal disease, some serotypes (and therefore their parent strains) are much more pathogenic than others. In addition, antibiotic resistance is closely tied to strain and as a result, some strains are harder to treat. There has been extensive work in this area of pneumococcal research, and in the revised version of the manuscript, we have added some examples to the introduction and discussion.**

5) What proportion of people are healthy/asymptomatic carriers? Is there any point to vaccination if rare NVT strains are just going to take the place of VT strains post-vac? Do we need new vaccines (e.g. each booster dose has a different combination of strains)? Will immigration of VT strains from unvaccinated individuals re-establish dominance post-vaccination? What is the impact of the polysaccharide vaccine on strain prevalence? Since the PCV is only administered to children and polysaccharide vaccine to seniors, how do middle aged individuals contribute to strain diversity? Essentially, why is forecasting the evolution of Streptococcus pneumoniae important? This is briefly touched on in a hypothetical manner in the discussion (lines 325-328), but per Plos Bio reviewer guidelines it needs significant work on explaining the 'novelty and significance of the findings'.

**The proportion of the population that carries pneumococcus asymptomatically varies by age and geography. In the US it is typically around 30% of children <6, but in Sub Saharan Africa can be much higher (>50%) and in all age groups. The question the reviewer asks about the point of vaccination is at the heart of the literature on ‘serotype replacement’. This has been an area of study for the last couple of decades, but up until now has been almost exclusively observational. The vaccines have had substantial net benefit, as they have reduced exposure to the virulent serotypes they target, although there remain impacts on older age groups that are not well understood (Lewnard and Hanage LID).**

**We intend for our study to have broad interest in the field of population genomics beyond pneumococcal researchers. In an effort to reduce the field-specific jargon, we omitted some details, which in fact may be of importance to the reader. This included detailed review of pneumococcal vaccinology. It is evident that this has led to a gap in the manuscript. We have since revised the introduction and discussion to include more information about the pneumococcal vaccines and epidemiology. The second paragraph of the introduction now contains much of the information requested by the reviewer: “*In the US, the PCV7 vaccine was introduced in 2000 and targeted seven serotypes (4, 6B, 9V, 14, 18C, 19F, and 23F) that were responsible for the greatest proportion of invasive pneumococcal disease, while the PCV13 vaccine was introduced in 2010 and targeted 13 serotypes (1, 3, 4, 5, 6A, 6B, 7F, 9V, 14, 18C, 19A, 19F, and 23F) [cite]. Following widespread introduction of PCVs, the carriage prevalence of pneumococci remained largely unchanged (~30% of the general population) [12] and non-vaccine serotypes (NVT) benefitted from the removal of their vaccine-serotype (VT) competitors and became more common in carriage and disease, with the gains from reducing VT disease partly offset by increases in NVT disease [13–15]. In some instances, this led to an increase in NVT serotypes that had increased virulence or harbored genes conferring antimicrobial resistance [16,17].*”**

**Further, we have highlighted the significance of the method and our findings in the introduction and discussion. In particular, we articulate one example with serotype 19A that had our method been employed prior to the development of PCV7 would have led to the inclusion of that serotype in the vaccine, likely reducing significant morbidity and mortality.**

*General comments*:

- I'm not familiar with PCV's, does the vaccine strain composition change over time? Or did it change over your study period, 1998-2012? (not PCV7 to PCV13, but whether the strains differed)

**As noted above, this information has been added to the introduction.**

- Is the PCV similar to the polysaccharide vaccine? Are they composed of the same strains? I assume all these samples were collected in children, as the polysaccharide vaccine is used in adults?

**The pneumococcal conjugate vaccines (PCV) contain the capsular polysaccharides of the pneumococcal serotypes they include. This makes them more immunogenic than the Pneumococcal polysaccharide vaccine (PPSV) that is used in adults. PPV-23 does have overlap with the serotypes included in PCV, as the serotypes for both were selected based on their invasiveness and prevalence. Samples from this study were collected from children. In the revised version of the manuscript, we have included information on the serotypes component of each PCV vaccine in the introduction (lines 79-81).**

- line 123 - are these 35 strains representative of strains in the US/world? The authors mentioned 90+, but what is the typical composition, are these 35 the most common?

**In the revised version of the manuscript we have clarified that the 90+ refers to serotypes, which are distributed across the population, and that a strain may be comprised of multiple serotypes. Strains (or sequence clusters) are defined statistically based on nucleotide variation in the genome. Therefore, the clustering may change slightly based on the sample. It is also worth noting that there is no generally agreed upon definitions for the *Streptococcus pneumoniae* global population structure, and historically there are been several attempts to classify using traditional (e.g., MLST and clonal complexes) and pathogen-specific (e.g., PMEM) methods. Most recently, an attempt was made to assign strains to the global population structure; however, this is yet to be widely adopted. Overall, the dominant strains are widespread, while the frequencies of those strains in each location may vary. For example, when comparing the Southwest US dataset to the Mass dataset, two strains were observed in Massachusetts that were not observed in the Southwest US** **sample and two low frequency strains were present in the Southwest US sample and not in the Massachusetts dataset. To clarify this in the revised manuscript, the following sentence has been added: *“In general, while their frequencies vary markedly between locations, the dominant S. pneumoniae strains are widespread”* (lines 96-97).**

- line 123/Fig 1 - From my understanding, the PCV covers 8/35 strains present?

**PCV7 targets 7 serotypes while PCV13 add an addition 6 serotypes to what was included in PCV7. Strains may be vaccine type (VT) only, non-vaccine type (NVT), or a mixed (VT-NVT). In the SW US sample, there were 3 VT strains and 5 mixed VT-NVT strains as shown in Figure 1.**

- Figure 1 - Is the post-vaccine 04C likely due to vaccine failure?

**It may be that slight variation in the capsule locus led to vaccine evasion; however, we don't have clear data on it. Exploring deviations from the NFDS model we present is an area of interest for future research.**

- Figure 1 - Thoughts on why strain 23 increased in prevalence?

**This a great question, we suspect that it is related to the serotype 15B/C component that has observed an increase globally; however, there is no agreement on why this strain has been more fit in the post vaccine landscape.**

- Figure 1 - Why did the null model only find significant difference in SC-22 and SC-23, but not SC-21? All were mixed with little change pre- and post-vac.

**This a very good observation, there indeed was little change in all three strains. However, based on the VT composition, 22 and 23 were expected (in the pro-rata model) to decrease considerably after vaccination while 21 was expected to remain relatively unchanged (based on the proportion of VT and NVT isolates in each strain). Therefore, the deviations of 22 and 23 from the pro-rata model were significant while 21 was not.**

- Figure 1/S1 - Were the 3 previous studies performed on the entire population? Was the entire population vaccinated?

**The three studies in the SW US were all conducted in the exact same communities. The vaccine trials were cluster randomized at the community level and our “pre-vaccine” sample came from the control communities. This is detailed under the “Study Population” in the methods section.**

- line 130 - Shouldn't this be 14 years, not 12 years afterward? 1998-2012?

**We were referring to 12 years after the introduction of vaccine in 2000. We have clarified this in the text.**

- line 130 and last line of caption on Fig 1 - 13/33, because you didn't include SC-12 and SC-24?

**Yes, those were excluded for that part of the analysis. This has been clarified in the S2 Table in the revised manuscript.**

- line 701/702 - How has relative prevalence not change pre-vaccine to predicted fitness? The two bottom blue lines seem to have swapped positions?

**Relative prevalence in this context refers to the prevalence of each strain during each time period (pre-vaccine and post-vaccine). In the pre-vaccine period, this included three “vaccine-type” strains that were removed by the vaccine. The prevalence is then calculated based on the remaining strains, as was done with the empirical data. The strains represented by the bottom two lines have changed rank in prevalence from pre-vaccine to post-vaccine equilibrium.**

- Fig 2A - Why are there units of time on the x-axis? Since this is conceptual, wouldn't it be more sensible to label each period as an epoch (establishing equilibrium, pre-vac equil/steady state, vacc intro, pred fitness, est. equilibrium, post vac equil/steady state)?

**We thank the reviewer for this observation, the numbers in the x-axis in Fig 2A have been removed.**

- line 705 - any motivation by choosing 10 simulations? These look to be fairly noisy though the general result can be seen. Why not choose the 35 strains you have in your study, rather than 35 at random?

**Each simulation is characterized by 35 randomly chosen strains that have unique presence/absence composition of 2,371 genotypes, similar to the Southwest US dataset. We chose 10 simulations to highlight the robustness of our method, which is independent of a particular set of strains.**

- Fig 2B - Thoughts on why the model never incorrectly predicted an increase in fitness, but was only incorrect when predicting a decrease? Going back to Fig 1A, there are a few examples of this in the data where I would have expected to see an increase, yet there was a decrease (SC-06A, SC-06B, SC-19A, SC-07, SC-03B etc.)

**This is a great question, and unfortunately, we currently do not have an answer for this. However, this is an area of interest for future research.**

- line 179/Fig 3 - Why only test 31 strains here, rather than the 35 in all other examples? Are the 4 taken out the ones either not present pre- or post-vaccine? I think this is explained on line 718, but I can only see two strains (SC-10 and SC-24) without NVT isolates before vaccination, not 4.

**This has been clarified in the figure legend and included in the new S2 Table.**

- line 190 - There are 33 strains in Fig 1 pre-vaccine, why only 27? Caption on Fig 3 says post-vaccine, but there are 33 of those in Fig 1...

**This has been clarified in the figure legend and included in the new S2 Table.**

- Fig 3 - I'm a bit confused on strain selection. In Fig 1 there are 5 mixed composition strains all present pre- and post-vaccine. In Fig 3A there are 4 and 3B-3D there are only 2.

**See the above response regarding the strain selection for each analysis.**

- Even after reading the methods, the number of strains in each figure is still confusing.

**We hope that the reviewer finds the additional text in the methods section, Tables S1 and S2, and new figure S4 valuable information that helps to understand better the assumptions made in our analyses.**

- Fig 3A - Possible to include a legend indicating mixed vs NVT?

**In the revised version, we have added a legend to Fig 3A.**

- Fig 3A - possible to move label SC-03B to whitespace to improve legibility?

**In the revised version, we have moved the label for SC-03B.**

- line 723 'increase'

**This has been corrected.**

- line 728 - any reason to choose the 1.5x IQR for annotation?

**We had to establish a statistical cutoff and based on the distribution of prevalence differences and this value was appropriate.**

- line 730 slope, CI, etc. all with or without sample SC-09?

**That figure includes strain SC-09. Later, we performed a sensitivity analysis removing SC-09 to confirm that this single strain wasn’t driving the association.**

- line 206 - when was the first year of vaccination? After reading the methods, I see this, but it would be nice to have basic year information in the text.

**The Southwest US dataset is unique in that it was the site of PCV7 vaccine trials; therefore, all isolates were collected from a vaccine-free community, even if they were collected between 2000-2002. As stated in the methods “*The pre-vaccine sample was collected from the well-defined control communities of the group-randomized trial of the PCV7 vaccine. Pre-vaccine isolates included in our study were collected between March 1998 and April 2001. In late October 2000, PCV7 vaccination became routine, including catch-up for children aged <5 years. By March 2001, a total of 88% of 3–4-month old infants living in PCV7-randomized communities and 77% of those in control communities had received >1 dose of PCV7*”. The Massachusetts sample was from the general US population for which PCV7 was introduced in 2000. We have clarified in the introduction that PCV7 was introduced in the general population in the US in 2000.**

- line 208 - mentions 6 years is not enough time for evolution, whereas 12 is - evidence/citation? (more on this below) From the x-axis on Fig 2A it seems like 6 years would be plenty (if those are years) - another reason to remove time units from 2A.

**In our first study of the SW US sample, we assessed the post-vaccine population collected ~5-6 years after the introduction of vaccine and then again at the 12 year mark. Based on the distribution of the strains and the frequencies of accessory genes (Figure 4, Azarian, PLoS Pathogen, 2018), we determined that the population had not equilibrated. Similarly, when we compare the frequencies of the accessory genes between the “peri” and “post” periods in the Massachusetts dataset, they were similar to what was observed at the immediate post period in the Southwest US sample. Overall, that sample was collected after the vaccine was introduced and it takes years to achieve high vaccine coverage; therefore, we suggest that this observation period was not ideal. Unfortunately, it is the best that we have available at this time. Last, the x-axis of figure 2A is arbitrary and represents of iterations of the simulation. We have removed the units of the x-axis on Figure 2A in the new version of the manuscript.**

- line 213 - PCV13 was introduced in 2010, but lines 357-358 identify post-pcv7 as 2010-2012 which you say is 'post-vaccine sampling'.

**We have included below supplementary figure 3 from our PloS Pathogens article. It addresses your question more directly.**

**PCV13 vaccine coverage, proportion PCV13 VT, and isolate sampling by month and year during Epoch 3 (2010–2012). Reservation-wide PCV13 vaccine coverage (primary y-axis–black) was obtained from Indian Health Services data. Coverage is based on three doses in children 7-<12 months or 1+ dose in children from 12–59 months. The proportion of PCV13 VT carriage in children <5 years of age was calculated using data from the PCV13 parent study (n = 1,603) (primary y-axis–red). The bar-plot represents the collection month of isolates used in Epoch 3 of the present study (secondary y-axis–grey).**

- line 313 - I think immigration is important here. You were able to show massive difference pre- and post-vaccine, but with re-introduction of VT strains (e.g. SC-17 and SC-12) through individual movement/transmission, will they increase in prevalence again?

**Immigration would allow for the importation of NVT strains into the communities that have high vaccine coverage. If these NVT strains are ideal replacements (based on genotype and NFDS dynamics), then we would expect them to increase. This is why it is important to prospectively sample from all populations and apply the method we describe here to assess their invasion potential.**

- line 235 - is there already work on invasive capacity of various strains?

**Yes. There is extensive work on variation in invasive capacity by serotype. We have added one such citation to the part of the discussion: Yildirim, I., Hanage, W.P., Lipsitch, M., Shea, K.M., Stevenson, A., Finkelstein, J., Huang, S.S., Lee, G.M., Kleinman, K. and Pelton, S.I., 2010. Serotype specific invasive capacity and persistent reduction in invasive pneumococcal disease. *Vaccine*, *29*(2), pp.283-288.**

- line 328 - are there less-virulent strains?

**Yes. The capsular polysaccharide, which determines the serotype, is the main virulence determinant and there is tremendous variation in invasive capacity. We have clarified the text to state this.**

- line 328 - even if we selected for drug susceptible lineages, wouldn't non-susceptible lineages pop up (per your results)?

**While antibiotic resistance determinants are often harbored on mobile elements (transposons, plasmids, phages), previous work has shown that those elements remain stable within the strain and population. Indeed, resistance can jump to other strains, creating new genotypes; however, our primary concern work be selecting for existing strains that are widely distributed that already possess antibiotic resistance determinants. Either way, our approach at gives us, at the least, some insight into what will happen to the population after the introduction of the vaccine.**

- line 371 - gene frequencies are dynamic by nature, especially in small sample sizes - are there time series showing stable gene frequencies in small populations without vaccination? Fig S3 from the plos path paper is as likely to be noise as it is perturbation on its way to equilibrium.

**We apologize, but we are unclear which aspect of the data the reviewer is referring to. In the PloS Path paper, Figure S11 shows the scatterplots of accessory genome loci across the various time points (E1-E3). We feel that drilling down to more local geographies would introduce more noise due to variations in strain distribution and frequency. To address this question, it would likely require extensive sampling from each location at multiple time points, which does not currently exist in a longitudinal population genomic sample. In the present revised version of the manuscript, we have added a paragraph on bacterial recombination that discusses how gene frequencies and strains vary over time.**

**Reviewer #3:**

1) Even though the authors say there is not much difference in terms of geography, I would like to see that in a plot and some statistics confirming that. My gut feeling is that immigration will pay a role in diversity.

**We are not sure what the reviewer is referring to – this could be interpreted as the geographic differences among communities in the Southwest US sample or between the Southwest US and the Massachusetts sample. We have detailed in the responses to the reviewer comment above (Reviewer #1, comment (1)) about the distribution of strains globally. The geographic relatedness of strains is further detailed by Corander and colleagues (Nat Ecol Evol, 2017, doi:10.1038/s41559-017-0337-x). Given this was not raised by the other two reviews we respectfully ask that we allow the manuscript to stand as it is in this regard.**

2) Also, I would like to see discussion on redundancy of some of these accessory genes and how that might affect predictive power.

**We have added the following sentence at Lines 320-322 of the discussion,** ***“…, as components of the accessory genome may be redundant, meaning they encode similar functions, or represent a single mobile element, our predictive power may be limited. Nevertheless, given the many potential pressures, mostly not directly observable, that we might expect to structure the pneumococcal population it is notable how effectively this approach can predict the impact of this perturbation.”* In addition, the newly added paragraph on recombination discusses the linkage among accessory genes.**

3) Finally, a paragraph on recombination and how that affects lineage composition would be appropriate.

**In the revised version of the manuscript, we have added a paragraph in the discussion (lines 276-292) on bacterial recombination, which also discusses lineage composition. In addition, we have added information on the age of lineages/strains in the sample as well as the global distribution of strains to address the representativeness of our sample.**
