# Supplementary material for: Frequency-dependent selection can forecast evolution in Streptococcus pneumoniae
Source: PLoS Biol. 2020 Oct 22;18(10):e3000878. doi: 10.1371/journal.pbio.3000878 (PMC7580979; doi:10.1371/journal.pbio.3000878)
Supplement: Attachment — Submitted filename: Reviewer_response_Final.docx [file pbio.3000878.s013.docx]

**Regarding data availability**

We have included the raw data and underlying code as supplemental material in the revised submission.

**Reviewer #1: [identifies himself as Chris Illingworth]**

*Minor comments:*

*I am happy with the response made to previous comments.  For clarity I suggest in line 417ff. using the notation x_i^{post} and x_i^{pre} rather than 3 and 1.*

We thank the reviewer for their subsequent review and the suggestion at line 417ff. We have made the suggested change in the revised manuscript.

**Reviewer #2: [identifies himself as Kevin Bakker]**

*This version of the manuscript is much improved. The additional text and figures (Particularly Fig S4, Table S2, and small changes/additions to the methods and discussion) helped my understanding of the samples, periods, and motivation. The authors addressed all of my major concerns and I only have a minor comment below.*

Thank you for your review and comments. They have significantly improved the manuscript and expanded its readability.
